# Advanced targeted nanomedicines for vulnerable atherosclerosis plaque imaging and their potential clinical implications

**DOI:** 10.3389/fphar.2022.906512

**Published:** 2022-10-13

**Authors:** Xue Li, Menglin Wu, Jiang Li, Qi Guo, Yang Zhao, Xuening Zhang

**Affiliations:** Department of Radiology, Tianjin Medical University Second Hospital, Tianjin, China

**Keywords:** atherosclerosis, vulnerable plaques, atherosclerosis molecular imaging, targeted nanoparticle-based contrast agents, imaging biomarkers

## Abstract

Atherosclerosis plaques caused by cerebrovascular and coronary artery disease have been the leading cause of death and morbidity worldwide. Precise assessment of the degree of atherosclerotic plaque is critical for predicting the risk of atherosclerosis plaques and monitoring postinterventional outcomes. However, traditional imaging techniques to predict cardiocerebrovascular events mainly depend on quantifying the percentage reduction in luminal diameter, which would immensely underestimate non-stenotic high-risk plaque. Identifying the degree of atherosclerosis plaques still remains highly limited. vNanomedicine-based imaging techniques present unique advantages over conventional techniques due to the superior properties intrinsic to nanoscope, which possess enormous potential for characterization and detection of the features of atherosclerosis plaque vulnerability. Here, we review recent advancements in the development of targeted nanomedicine-based approaches and their applications to atherosclerosis plaque imaging and risk stratification. Finally, the challenges and opportunities regarding the future development and clinical translation of the targeted nanomedicine in related fields are discussed.

## Introduction

Atherosclerosis is the most frequent pathological substrate underlying ischemic heart disease or ischemic stroke, which has been a massive public health problem in China (Yang et al., 2016; Zhao et al., 2019; Libby, 2021). An epidemiology study on atherosclerotic cardiovascular disease (ASCVD) indicates that acute cardiovascular events caused by vulnerable plaque rupture show a rapidly and substantially increased mortality from 11% in 1990 to 25% in 2016 in China (Zhao et al., 2019). Given that atherosclerosis is a progressive disease that may remain asymptomatic for several years, early detection and treatment is the most useful strategy to prevent an unheralded rupture of atherosclerotic plaque. To effectively detect the appearances of risk factors of vulnerable plaques, non-invasive diagnostic modalities which can provide long-term and reproducible assessment with minimum risk are critically needed.

Current clinical non-invasive imaging modalities for stratifying the risk of atherosclerotic plaques include magnetic resonance imaging (MRI), computed tomography (CT), positron emission tomography (PET), single photon emission computed tomography (SPECT), and ultrasound imaging (US), depending on quantifying the percentage reduction in luminal diameter (Group et al., 2017). For example, according to the degree of luminal stenosis, the grade of atherosclerotic plaques is divided into mild (<50% in stenosis), moderate (50%–69% in stenosis), and severe (70%–99% in stenosis). Only severe stenosis is regarded as risk plaque. Nevertheless, a large number of acute vascular events arise from mild stenosis (<50% stenosis) rather than severe stenotic plaques (Naghavi et al., 2003). According to a study reported by Hyafil et al. (2016), there are more than 20% of carotid arteries with mild stenosis (<50% stenosis) exhibiting high-risk features of rupture, and about 8%–9% of carotid arteries with normal lumen size are found to have vulnerable features. Such nonstenotic vessels without any prior symptoms are probably the most neglected but risk plaques, causing unheralded devastating consequences such as stroke or acute myocardial infarction. In this regard, both the American Society of Neuroradiology (ASNR) and the European Society of Cardiology (ESC) propose the risk of carotid vulnerable plaque rupture should not only be attribute to the degree of stenosis but also to plaque compositions (Aboyans et al., 2018; Saba et al., 2018). Consequently, the diagnosis of high-risk atherosclerotic plaque, more than stenotic one *via* the non-invasive imaging technology, has become an area of intense research studies. Although plentiful imaging strategies for exploiting vulnerable features have been developed, it still remains a huge challenge to provide a more comprehensive and accurate assessment of high-risk atherosclerotic plaques.

With rapid development in nanotechnology and nanomedicine, there has been great potency in using nanoparticles for medical imaging. Nanoparticles are one kind of particles in the range of 1–1,000 nm in size. Owing to the unique properties conferred by their nano-scale size and modular structure, nanoparticle fabrication can be precisely controlled, allowing their physical characteristics, such as shape, size, surface charge, and biodegradability, to be modified as required (Lobatto et al., 2011). On this basis, nanoparticles can serve as efficient imaging contrast agents to tremendously enhance imaging contrast *via* incorporating or labeling with a great deal of imaging motifs, including Gd^3+^ and Fe^3+^ for MRI; Au for X-ray and CT imaging; ^64^Cu for PET and ^111^In for SPECT imaging; fluorophores and quantum dots for optical imaging (Hu et al., 2020; Shi et al., 2020; Chen et al., 2021). More importantly, nanoparticles have high a surface-to-volume ratio, allowing the surface layer to be modified with antibodies, proteins, peptides, or other ligands which can target single or multiple receptors overexpressed on the surface/inside of atherosclerotic plaques (Guo et al., 2021). Therefore, the nanoparticles can be designed as novel imaging platforms to diagnose atherosclerosis at the molecular level (Guo et al., 2021). This review aims to provide a fresh perspective on an overview of the various targeted nanoparticle-enhanced imaging strategies for vulnerable plaque identification, along with the perspective of their distinct functions and current challenges in distinguishing the vulnerable from the stable atherosclerotic plaques, as well as their performance beyond traditional imaging modalities.

## Key processes in atherosclerosis development

**SCHEME 1:**
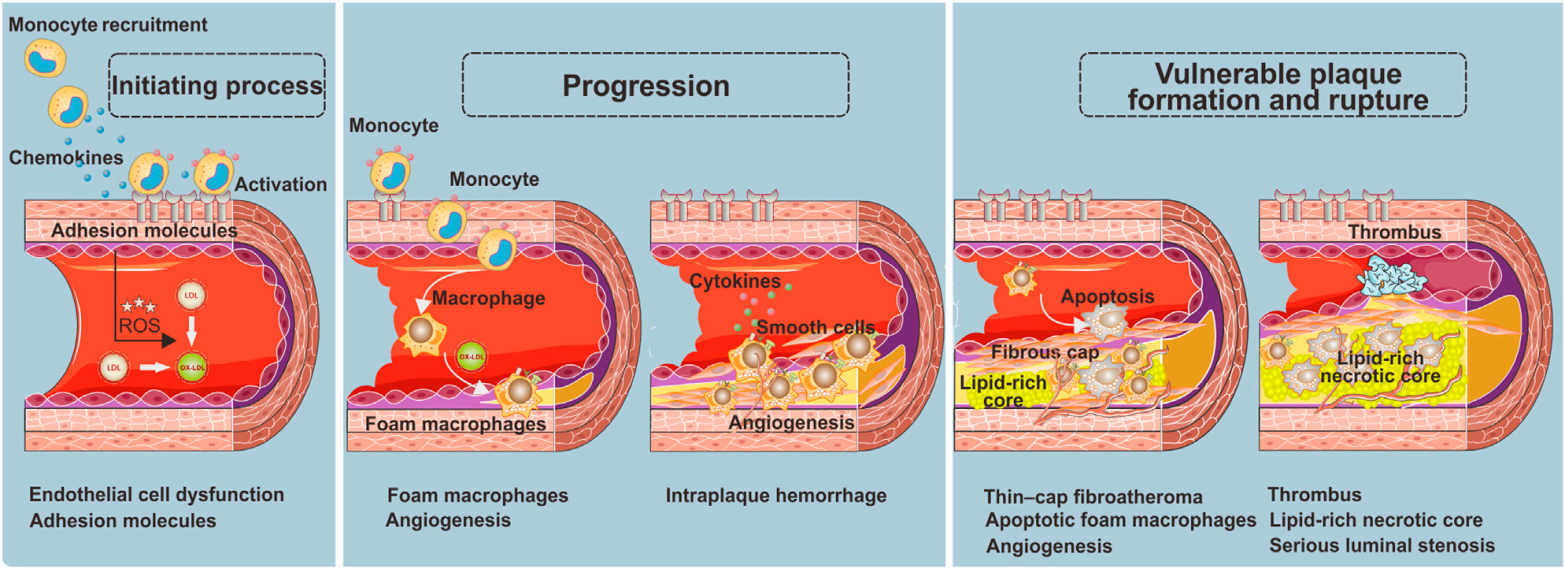
Key processes of the atherosclerotic plaque development.

Atherosclerotic plaques are regarded as a gradual buildup of a heterogeneous collection of lipids, fibrous tissue, inflammatory cells, and other materials in the arterial wall. This process involves a series of specific cellular and molecular events in the arterial lesions, which can serve as local targeting epitopes for nanoparticles (Shah, 2009; Magnus et al., 2019), including endothelial cell activation (Andelovic et al., 2021), inflammation (Soehnlein and Libby, 2021), angiogenesis (Camaré et al., 2017), apoptosis (van Tilborg et al., 2010), platelet activation, and thrombus (Sanz and Fayad, 2008) (Scheme 1).

### Endothelial cell dysfunction as the initiating process in atherosclerotic plaques

Endothelial cells, the inner layer of the arterial wall, perform a vitally important regulatory function in controlling the vascular permeability of macromolecules *via* dynamic intercellular gaps, intracellular fenestrae, and vacuolar pathways initiated in endocytic vesicles (Chiu and Chien, 2011). Endothelial cell dysfunction is a crucial pathophysiological factor causing atherosclerosis (Tabas et al., 2007), which results in defective endothelium, creates the pro-inflammatory state, and increases the expression of cell adhesion molecules (e.g., intercellular adhesion molecule 1, ICAM1; vascular cell adhesion molecule 1, VCAM1; E-selectin and P-selectin). The defective endothelium further induces neutrophils and monocytes to transmigrate into the arterial wall, which would perpetuate the local inflammatory response by secreting chemokines (Boring et al., 1998; Nahrendorf et al., 2006; Kwon et al., 2018). As a result, low-density lipoprotein (LDL) and cholesterol particles transendothelially penetrate and stay in the subendothelial space, contributing to the lipid disorder and exacerbation of inflammation (Tabas et al., 2007). Thus, endothelial cell dysfunction is regarded as a certain feature for detection of early or still-reversible atherosclerosis (Rucher et al., 2019; Tiwari et al., 2021).

### Foam macrophages as the progression of atherosclerotic plaques

After migrating into the arterial vessel wall, monocytes differentiate into macrophages and further polarize into pro-inflammatory lesioned macrophage phenotypes, causing the initially intimal lesion (also called fatty streak) (Moore and Tabas, 2011). At this stage, these pro-inflammatory macrophages express scavenger receptors to facilitate the ingestion of oxidized-LDL cholesterol particles (ox-LDL) and transformation to foam macrophages. The appearance of substantial foam macrophages is considered a key feature of the plaque progression from a stable to unstable stage. The scavenger receptors, including macrophage scavenger receptor 1 (SR1), CD36 receptor, and lectin-like oxidized receptor (LOX-1), are thereby supposed to be the ideal markers of plaque instability (Stöger et al., 2012; Bentzon et al., 2014). Notably, in addition to macrophages, vascular smooth muscle cells (VSMCs) also play a considerable role in foam macrophage formation, referred to as VSMC-derived foam cells (Allahverdian et al., 2014). Consequently, the proteins that have direct atherogenic effects on VSMC, for example, an intracellular actin-binding protein called profilin-1, are regarded as available targets of foam macrophages for unstable plaques.

In addition to the accumulation of foam macrophages, neovascularization is another vital feature of atherosclerosis progression (Moreno et al., 2006). As atherosclerosis progresses, the neointimal will become thicker. Once the thickness between the neointimal and luminal surface exceeds the oxygen diffusion threshold, local hypoxia and neovascularization will occur. It is well known that the neovascularization would further promote the pro-inflammatory monocyte recruitment and lipoprotein deposition, exacerbate plaque burden and vulnerability, and increase intraplaque hemorrhage (Kolodgie et al., 2007; Combadiere et al., 2008).

### Formation and rupture of vulnerable plaques

Continuous accumulation of ox-LDL and pro-inflammatory monocytes gravely amplifies the local storage of foam macrophages, and inevitably, a portion of apoptotic foam macrophages cannot be removed by efferocytosis effect in time. These secondary necrotic foam macrophages comprise the necrotic lipid core, which is the most important indicator of the formation of vulnerable plaques, signifying the stepwise progression of plaques toward a more vulnerable stage (Virmani et al., 2006; Yurdagul et al., 2020). To stabilize the fragile plaques, a fibrous cap containing migrating smooth muscle cells, fibroblasts, as well as extracellular matrix including collagen will cover the surface of the necrotic lipid core. Nevertheless, as the inflammation within the plaque is in progress, the fibrous cap will become thinner and even break down to cause thrombotic occlusions and clinical events. Since then, a large lipid-rich necrotic core, thin-cap fibroatheroma, neovascularization, thrombus, and serious luminal stenosis are considered the major features of the vulnerable phenotype (Sanz and Fayad, 2008). In this regard, both morphological and pathophysiological imaging information are equally important for non-invasive identification of the plaques with vulnerable or rupture-prone characteristics.

The aforementioned processes and the accompanying molecular and cellular events aggravating the atherosclerotic plaque provide a plenty of compelling targets for nanoparticle-assisted diagnosis of atherosclerosis. Interestingly, the microenvironmental conditions of atherosclerotic lesions, such as endothelial dysfunction, upregulated adhesion molecules, acidic pH, hypoxia, neovascularization, and inflammatory, are to some extent similar to tumors (Lobatto et al., 2011). Thus, the key targeting imaging principle used in a tumor is a promising strategy for atherosclerosis. For example, analogous to tumors, with the assistance of an immature lymphatic drainage system, dysfunctional and leaky blood vessels of atherosclerotic plaque allow the nanoparticles to locally accumulate at the plaque site through the enhanced permeability and retention (EPR) effect (Barua and Mitragotri, 2014). However, there are still several existing intrinsic discrepancies between tumor and atherosclerosis that should be notified: 1) atherosclerosis is a systemic vascular disease rather than a focal disease; thus, the plaques may simultaneously develop at the lumen site with more than one lesion; 2) a high degree of overlap exists between inflammatory-related mechanisms involved in atherosclerosis and host defense, which makes the inflammatory mechanism between lesions and normal host defense hard to distinguish, increasing the difficulty of precise targeting. Under these conditions, new targeting strategies to specifically and effectively reflect the real plaque biology and pathophysiology are needed for atherosclerosis imaging.

## Mechanistic aspect of nanoparticles homing to atherosclerotic lesions

### Basic principles of nanoparticle-based imaging strategies

Rapid advances in nanotechnology greatly accelerate the development of novel nanoparticles for visualization of atherosclerotic plaques at high risk. In contrast to conventional small molecular contrast agents, engineered nanoparticles reveal notable advantages in substantially augmenting the accumulation of imaging contrast at plaques due to their prolonged blood circulation and EPR effect. Moreover, after being labeled with binding ligands, nanoparticles can specifically target and visualize the key processes that aggravate the progression of atherosclerotic plaques, providing pivotal insights into plaque biology. Generally, nanoparticle-based contrast agents typically include a contrast-generating material to provide imaging information, and a targeting motif with high affinity to target the desired compositions. Thus, the corresponding imaging strategies can efficiently combine conventional cross-sectional imaging with molecular imaging together. Nowadays, diversity of nanoparticle-based imaging contrast agents has been explored to assess the composition of atherosclerotic plaque, quantify the atherosclerosis burden, and evaluate the efficacy of therapies at the molecular level (Table 1).

**TABLE 1:** Diversity of nanoparticles used in imaging of atherosclerotic plaques.

Nanoparticle platform	Imaging modality	Animal model	Application
Polymeric nanoparticles			
Polyglucose nanoparticles	PET/MRI	ApoE^−/−^ mice fed a high-fat diet; New Zealand White rabbits underwent double balloon injury of the thoracic and abdominal aorta	Non-invasively monitoring macrophage biology Keliher et al. (2017)
Hyaluronan (HA)-ATV nanoparticles	MRI	ApoE^−/−^ mice fed a high-fat diet	Assessing the treatment effect of HA-ATV NP Nasr et al.(2020)
Semiconducting polymer nanoparticles	PAI	ApoE^−/−^ mice fed a high-fat diet	Evaluating the inflammation level of atherosclerosis Ma et al.(2021)
Biomimic materials			
Platelet membrane-based nanocomplexes	MRI	ApoE^−/−^ mice fed a high-fat diet	Assessing the development of atherosclerosis Wei et al.(2018)
Human ferritin cages	Fluorescence/MRI	FVB mice with high-fat diet and streptozotocin injections	Imaging atherosclerotic carotid Terashima et al.(2011)
^99m^Tc-labeled human ferritin cages	PEI	ApoE^−/−^ mice fed a high-fat diet	Imaging multiple high-risk features of macrophage infiltration, active calcification, positive remodeling, and necrosis Liang et al.(2018)
HDL nanoparticles	MRI	ApoE^−/−^ mice fed a high-fat diet	Assessment of anti-inflammation therapy Duivenvoorden et al.(2014)
Inorganic nanoparticles			
USPIO	MRI	Human	Imaging inflammation level of atherosclerosis Kooi et al.(2003), Tang et al.(2009), Zheng et al.(2019)
Mesoporous silica-coated iron oxide nanoparticles	MRI	ApoE^−/−^ mice fed a high-fat diet	Evaluating the macrophage level of atherosclerosis Wu et al.(2021)
Gd complex-containing nanoparticles	MRI	ApoE^−/−^ mice fed a high-fat diet	Evaluating the macrophage level of atherosclerosis Wang et al.(2019)
Gd inorganic nanoparticles	MRI	New Zealand White rabbits underwent double balloon injury	MR angiography and atherosclerotic plaque imaging Xing et al.(2014)
Gold-coated iron oxide nanoparticles	MRI	ApoE^−/−^ mice fed a high-fat diet	Targeting CD163-expressing macrophages to detect the state of the atheromatous lesions Tarin et al.(2015)
Au nanoparticles	Multicolor CT	New Zealand White rabbits underwent double balloon injury	Quantifying the macrophage burden Cormode et al.(2010) and Si-Mohamed et al. (2021)
Upconversion nanoparticles	Fluorescence/MRI	C57 mice with high-fat diet and perivascular cuff placement	Visualization of vulnerable atherosclerotic plaque progression Wang et al.(2019)
Quantum dot-iodinated oil nanoemulsion	Fluorescence/CT	New Zealand White rabbits underwent double balloon injury	Visualizing atherosclerotic plaques Ding et al.(2013)

### Journey of nanoparticles to atherosclerotic plaques

Intravenous administration is the most effective delivery route for systemic vascular diseases. However, nonspecific clearance from the blood circulation usually occurs before nanoparticles reach the designed site, resulting in a low bioavailability of nanoparticles (Barua and Mitragotri, 2014). One of the pivotal mechanisms responsible for this rapid clearance is that the administrated nanoparticles usually absorb opsonin proteins, which can induce the undesired endocytosis by the mononuclear phagocyte system (MPS, e.g., liver and spleen) (Chen et al., 2017; Fleischmann and Goepferich, 2021). To evade the MPS recognition and clearance, nanoparticles are engineered to be protected with hydrophilic polymers [e.g., poly (ethylene glycol) (PEG)] (Zhang et al., 2021) or biomimetic materials, for example, red blood cell membranes (Wang et al., 2019), platelet membranes (Wei et al., 2018; Li et al., 2021), extracellular vesicles (Ma et al., 2020), macrophage-derived exosomes (Shan et al., 2021), high-density lipoprotein (HDL) (Lameijer et al., 2018; Binderup et al., 2019), and protein nanocages (Terashima et al., 2011; Liang et al., 2018). In addition, the physicochemical characteristics of nanoparticles such as the particle size, composition, and surface potential also contribute to determining the biological fate of nanoparticles. For example, when the nanoparticle size is comparable to the vascular fenestration of the liver (50–100 nm) or the inter-endothelial cell slits of the spleen (200–500 nm), a corresponding serious liver or spleen accumulation will occur (Maldiney et al., 2014; Du et al., 2017). A useful example in this term is a study reported by Song et al. (1998). They fabricated poly (D, L-lactide-co-glycolide) (PLGA, an FDA-approved material) nanoparticles with different sizes, showing that the PLGA nanoparticles with 100 nm size had 3-fold higher endocytosis than nanoparticles with 275 nm size in atherosclerotic plaques (Song et al., 1998). In a very detailed study, Tang et al. (2016) demonstrated the phospholipid contained HDL-mimicking nanoparticles presenting spherical morphology with 30 nm, exhibiting a notably prolonged blood half-life and optimal increased accumulation in aortic plaques.

After escaping from the MPS capture, nanoparticles should penetrate through the leaky vasculature into the vessel wall to reach the atherosclerotic lesions. Similar to tumors, the permeation pathway of nanoparticles penetrating into plaques is mainly dependent on the disrupted endothelial barrier (Kim et al., 2014). However, simply through the permeation pathway, nanoparticles can only accumulate in the peripheral areas of the plaque, leading to heterogeneous and inadequate deposition (Stein-Merlob et al., 2017). Otherwise, the permeation degree of nanoparticles may be critically dependent on the integrity of endothelial junction architecture. But in fact, in some cases, the endothelial junction architecture would be remodeled and become integral with the progression of atherosclerotic plaque, resulting in decreased perfusion of nanoparticles. As an example reported by Beldman et al. (2019), the group constructed hyaluronic acid-modified nanoparticles (HA-NPs) to investigate the permeation of nanoparticles in early and advanced plaques *in vivo*. They found the astonishing results: as leaky vasculature was the single pathway of nanoparticles to enter the plaque, the accumulation of HA-NPs in advanced plaques was about 3-fold lower than that of early counterparts (Beldman et al., 2019). In this paradigm, targeting functionalized nanoparticles that can enter the plaque lesions by permeation, and active targeting is particularly advantageous for addressing these challenges (Barua and Mitragotri, 2014).

Targeting functionalized nanoparticles can be internalized by a wide range of cell types in the plaque microenvironment. Therefore, cellular and molecular processes involved in atherosclerosis are able to serve as local targeting epitopes to enhance the nanoparticle internalization. For instance, VCAM-1, a vial component of the leukocyte–endothelial adhesion molecule, is the most prevalent adhesion molecule in atherosclerosis (about 82%) (Thayse et al., 2020). Given that the expression of VCAM-1 is correlated with early vulnerable plaque, Kelly et al. (2005) developed magnetofluorescent nanoparticles modified with the VCAM-1-targeted peptide (VHSPNKK) for MRI and fluorescence imaging of atherosclerotic lesions. Benefiting from the high affinity of the VCAM-1-targeted peptide, activated endothelia of atherosclerotic lesions were detected in *in vivo* MRI and fluorescent imaging. Compared with the VCAM-1 monoclonal antibody, VHSPNKK peptide exhibited 12-fold higher target-to-background ratios. Another VCAM-1-targeting peptide (NNSKSHT) was reported to be conjugated on ultra-small superparamagnetic iron oxide nanoparticles (USPIOs) for the detection of early and advanced atherosclerotic lesions in ApoE^−/−--^ mice (Michalska et al., 2012). The corresponding MR images showed that the VCAM-1-overexpressed endothelial cells were visualized *in vivo* with the targeting USPIOs. Certainly, in addition to the adhesion molecules, other ligand–receptor systems related to macrophages, annexin V, protease activity, angiogenesis, thrombosis, fibrin, and platelets have also been comprehensively investigated, as described in Table 2.

**TABLE 2:** Diversity of current ligand–receptor systems.

Process	Target	Ligand	Format	Application
Endothelial disorder	VCAM-1: vial component of the leukocyte–endothelial adhesion cascade that correlated with the extent of exposure to atherosclerotic risk factors	VHSPNKK	Peptide	Detection of activated endothelium of atherosclerotic lesions with MRI and optical imaging Kelly et al.(2005) and Michalska et al.(2012)
	VCAM-1	VHPKQHR	Oligopeptide	Detection of atherosclerotic plaques by MRI and optical imaging dual-model Nahrendorf et al.(2006)
	VCAM-1	Cyclical (NNSKSHT)	Cyclic peptide	Detection of early and advanced atherosclerotic lesions in ApoE^−/−^ mice by MRI Michalska et al.(2012)
	VCAM-1	scFvm_VCAM-1_	Single-chain antibody	Potential theranostic nano-delivery systems to downregulate VCAM-1 expression and longitudinally evaluate therapeutic efficacy Wang et al.(2018)
	ICAM-1: mediators of leukocyte migration from the blood vessel into the endothelium and intima.	Anti-ICAM-1 single-domain antibodies (sdAb)	Antibody	Combination of morphological and biological biomarkers to identify atherosclerotic plaques using real-time intravascular bimodal IVUS-NIRF imaging catheter Bertrand et al. (2019).
	ICAM-1	Anti-ICAM-1 antibody	Antibody	Early detection of atherosclerotic plaques by CT imaging Danila et al.(2009). Detection of atherosclerotic plaques and assessment of inflammation-related ICAM-1 expression by MRI Paulis et al.(2012)
	ICAM-1	NNQKIVNLKEKVAQLEA Garnacho et al.(2012)	Peptide	—
	CD81: a ubiquitously expressed tetraspanin upregulated in the endothelium of atherosclerotic plaques in human artery protein	Anti-CD81-antibody	Antibody	Early detection of atherosclerotic plaques by MRI Fei et al.(2015)
	Bovine aortic endothelial cells (BAECs): specific to aortic endothelial cells activated by TNF-alpha and LPS	CLWTVGGGC Thapa et al.(2008)	Peptide	—
Inflammatory cells	Monocyte C–C chemokine ligand 2 (CCR2): overexpression on the surface of monocyte chemoattractant protein-1	YNFTNRKISVQRLASYRRITSSK	Peptide	Detecting and discriminating various stages of atherosclerosis Chung et al.(2014)
	Monocyte C–C chemokine receptor 5 (CCR5): an active participant in the late stage of atherosclerosis	ASTTTNYT	Peptide	Imaging the expression of the CCR5 receptor with PET/CT in a wire-injury-induced ApoE^−/−^ model Luehmann et al.(2014)
	Macrophage scavenger receptor 1 (MSR1): macrophages play key roles in atherosclerosis progression	Anti-MSR1 (anti-CD204) monoclonal rat anti-mouse antibody	Antibody	Assessment of macrophages content in atherosclerotic plaque using MRI *in vivo* Amirbekian et al.(2007)
	CD36: a class B macrophage scavenger receptor which play an important role in oxidized lipoprotein uptake.	Haic-D-2MeTrp-D-Lys-Trpe-D-Phe-LysNH2	Peptide	Elicits macrophage-to-feces reverse cholesterol transport in a manner dependent on CD36 expression Bujold et al.(2013)
	CD36	Anti-CD36 antibody	Antibody	MR detection and characterization of atherosclerosis plaque Lipinski et al.(2009)
	Lectin-like oxidized low-density lipoprotein receptor 1: mediate the pathologic effects of oxLDL in atherosclerotic lesions	Anti-LOX-1 antibodies	Antibody	*In vivo* MR imaging of atherosclerosis plaque Wen et al.(2014)
	Scavenger receptor AI (SR-AI): overexpresses on foamy macrophages surface	LSLERFLRCWSDAPAK	Peptide	MR detection and characterization of high-risk atherosclerosis plaque Wang et al.(2019). Imaging of high-risk plaque in ApoE^−/−^ mice with *T*_*2*_ and *T*_*2*_ * mapping Wu et al.(2021)
	Apo A-I mimic	18A: DWLKAFYDKVAEKLKEAF37pA: DWL KAFYDKVAEKLKEAFPDWLKA FYDKVAEKLKEAF	Peptide	Comparison of the cholesterol efflux effect of two different apo A-I mimicking nanoparticles and assessment of their distribution and targeting ability *in vivo* imaging Cormode et al. (2009).
	Apolipoprotein E (apoE)	LRKLRKRLLR	Peptide	Detection of intraplaque macrophages that are associated with plaque vulnerability Wei et al.(2010)
	Folate receptor beta (FR): specifically expresses on activated macrophages	Folic acid (FA)	Small molecular	Delivery system for targeting chronically activated macrophages in atherosclerosis plaque Rollett et al.(2012)
	Phospholipid phosphatidylserine (PS): exposed on apoptotic cells, activated platelets, and activated macrophages	Annexin V	Protein	Delivery superparamagnetic contrast agents to sites containing apoptotic cells for atherosclerosis plaque imaging Smith et al.(2007). Evaluation of atherosclerotic plaque vulnerability by MRI van Tilborg et al.(2010)
	Matrix metalloproteinases (MMPs): activated cells in inflamed atherosclerotic plaques (e.g., macrophages, smooth muscle, and endothelial cells) produce MMPs. The concentrations under pathological stress and correlation with plaque vulnerability.	P947: Gly-Pro-D-Leu-D-Ala-NHOH	Peptide	Detection and characterization of the matrix metalloproteinase (MMP)-rich atherosclerotic plaques Ouimet et al.(2012)
	MMPs	GGPRQITAG	Peptide	Evaluation of the atherosclerotic plaques with activatable NIRF imaging Deguchi et al.(2006)
	Cathepsin K: exhibits the highest capability to degrade components of the extracellular matrix	Abz-HPGGPQ-EDN2ph Deguchi et al.(2006) and Lecaille et al.(2003)	Peptide	—
Angiogenesis	α_γ_β_3_ Integrins	Vitronectin	Protein	Noninvasive assessment of angiogenesis in early atherosclerosis, for site-specific delivery of antiangiogenic drug and for quantitative follow-up of response Neubauer et al.(2006)
Thrombus	Fibrin: a major constituent of a clot	GPRPP	Peptide	Visualization of the venous thromboembolism and pulmonary embolism *in vivo* by PET imaging Aruva et al.(2006)
	Fibrin	Anti-fab’ fragment	Antibody	Detection of thromboembolic events Macfarlane et al.(2009)
		Humanized monoclonal antibody fragment specific for the D-dimer region of cross-linked fibrin	Antibody	Assessment of suspected deep vein thrombosis Douketis et al. (2012).
		YQCPYGLCYIQ	Peptide	Detection of thrombus by MRI Overoye−Chan et al. (2008).
	Fibrin and thrombin	Cleavable ligand: KKLVPRGFibrin- targeting ligand: GPRPPGGS[Lys(TMR)]GC	Peptide	Detection of the stage of thrombosis in cardiovascular diseases Ta et al.(2018).
	Thrombin	Gly-D-Phe-Pip-Arg-Ser-Gly-Gly-Gly-Gly-Lys-Cys	Peptide	Imaging of thrombin activity *in vivo* Jaffer et al.(2002)
	Activated factor XIII (FXIIIa)	N_13_QEQVSPLTLLK_24_	Peptide	*In vivo* imaging of FXIIIa activity for detection of acute thrombi Jaffer et al.(2004)
	Membrane glycoprotein Ilb/IIIa (GPIIb/IIIa) receptor	(D-Tyr)-Apc-Gly-Asp (Apc: S-aminopropyl-L-cysteine)	Peptide(cyclic)	*In vivo* thrombus imaging Lister-James et al.(1996)
	GPIIb/IIIa	(D-Tyr)-Amp-Gly-Asp (Amp: 4-amidinophenylalanyl	Peptide(cyclic)	*In vivo* thrombus imaging Lister et al.(1997)
		CRGDC	Peptide(cyclic)	Identification of atherothrombosis and vulnerable plaques Klink et al.(2010)
		N-methyl-L-arginyl-glycyl-L-aspartyl Mousa et al.(1993)	Peptide(cyclic)	—
		Cyclo(D-Val-NMe-Arg-Gly-Asp-Mamb) Harris et al.(1996)	Peptide(cyclic)	—
		Anti-GPIIb/IIIa scFv	scFv	Targeted simultaneously *T*_*1*_ and *T*_*2*_-weighted imaging of thrombosis Ta et al.(2017)
Other targets	IL-4 receptor	CRKRLDRNC	Peptide	Detection of atherosclerotic with near-infrared fluorescence imaging Park et al.(2008)
	Stabilin-2: expression on macrophages, smooth muscle cells, and endothelial cells	CRTLTVRKC	Peptide	Homing to endothelial cells, macrophages, and smooth muscle cells of atherosclerotic plaques in Ldlr^−/−-^ mice Lee et al.(2011)

### Nanoparticles for molecular diagnostic imaging

In this section, we discuss the applications of nanoparticles for assessing vulnerable plaques by clinical and preclinical imaging technologies and emphasizing on how to improve the delivery efficiency and enhance the imaging contrast of nanoparticles. The relative advantages and limitations of each imaging modality for plaque detection are first compared and described in Table 3.

**TABLE 3:** Various non-invasive imaging modalities.

	MRI	CT	PET	UI	FI	PAI
Advantages	• High spatial resolution (μm)	• High spatial resolution (μm)	High sensitivity (pmol/L)	Short scan time (5–15 min)	• High spatial resolution (μm)	• Multiple wavelength scan
	• Excellent soft-tissue contrast	• Short scan time (5–15 min)			• High sensitivity (pmol/L)	• Short scan time (5–15 min)
	• High tissue penetration depth	• High tissue penetration depth			• Short scan time (1–5 min)	
		• 3D reconstruction				
Disadvantages	• Long scan time	• Low sensitivity	• Low spatial resolution (mm)	• Low spatial resolution (mm)	Limited tissue penetration depth	Limited tissue penetration depth
	• Low sensitivity	• Lack of specificity	• Lack of anatomical reference	• Lack of anatomical reference		
	• Lacking specificity			• Lack of specificity		
				• Limited tissue penetration depth		
Imaging agents	• Iron oxide nanoparticles	• Iodinated nanoparticles	• ^18^F-, ^89^Zr-, ^68^Ga-labeled nanoparticles	• Microbubbles	• Fluorescein-labeled nanoparticles	• Au nanoparticles
	• Gd-containing nanoparticles	• Au nanoparticles		• Nanobubbles	• Upconversion nanoparticles	• Near infrared dye-labeled nanoparticles
					• Quantum dot	
Morphological characterization of vulnerable plaque in clinical	• Endothelial permeability	Calcification	Lipid	Neovascularization	—	—
	• Intraplaque hemorrhage					
	• Inflammation activity					
	• Neovascularization					
	• Thrombosis					

## Magnetic resonance imaging

Due to its unlimited penetration depth, high temporospatial resolution, and excellent soft tissue contrast, MRI is regarded as one of the most prevailing imaging modalities for clinic and pre-clinic research studies (Gong et al., 2021). From an atherosclerotic plaque imaging perspective, MRI exhibits incomparable superiority compared with other imaging modalities. That is, MRI can be used with or without contrast agents to measure the luminal stenosis degree and crudely characterize vulnerable features by integrating multiple parameters into a single session, including the features of intraplaque hemorrhage, inflammation activity, intraplaque neovascularization, and thrombosis (Hatsukami et al., 2000; Saam et al., 2005; Hartog et al., 2012; Kim et al., 2017). Despite well-appreciated advantages, the inherent limitations of MRI, such as low sensitivity and inevitable image quality degradation, still severely hamper their applications in early detection and risk stratification of vulnerable plaque. In addition to that, the interpretations of MR signal patterns often highly rely on the radiologists’ expertise and experience, which may reduce the reproducibility or consistency of diagnostic results, impairing long-term monitoring of the atherosclerosis progression. In this regard, molecular MRI that can detect biological processes at the molecular level shows great potential to assess the plaque vulnerability. Given that most of vulnerable plaque targets are located in deep organs (e.g., aorta and coronary artery) with an extremely low concentration (nanomolar), nanoparticles with high sensitivity and specificity may be particularly suitable to be used as reliable contrast agents in molecular MRI.

**FIGURE 1:**
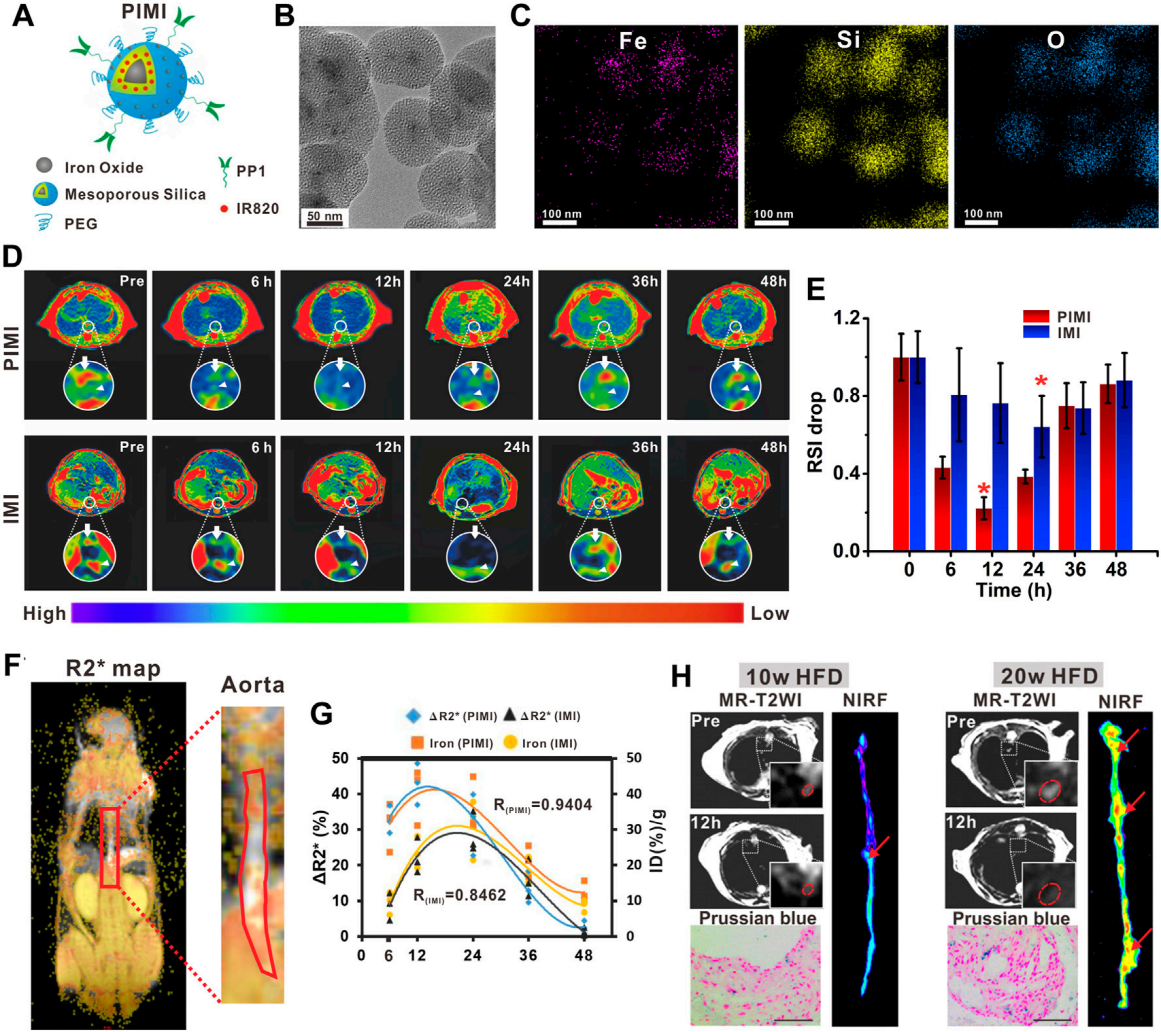
**(A)** Schematic illustration of PIMI. **(B)** TEM image and **(C)** elemental mapping images (Fe, Si, and O) of PIMI. **(D)** *T*_*2*_-weighted pseudo-color images after PIMI and IMI nanoparticle injection. **(E)** Relative MR signal intensity drop of aortic plaques at different time points. **(F)** Coronal R2* map of aorta. **(G)** Correlation of fitted curves between aortic R2* changes and nanoparticle deposition. **(H)** Dual-modal MR, NIRF imaging, and Prussian blue staining of plaques induced by 10 or 20 weeks of HFD. Scale bars: 50 μm (Copyright permission from Elsevier) (Wu et al., 2021).

Generally, the most commonly used nanoparticles for molecular MRI are categorized into *T*_*2*_-shortening agents based on iron oxide materials and *T*_*1*_-shortening agents based on Gd chelates. Both of these categories of nanoparticles can be modified to facilitate the uptake by specific cells, or fabricated with moieties with high affinity to the desired target molecules, thereby enhancing the local MR signals of interest components. To date, although the majority of nanoparticles for molecular MRI are still in the experimental phase, the commercial dextran-protected USPIOs, which are highly water-soluble with a particle size of 30–50 nm, have been used as the clinical *T*_*2*_ contrast agents for human atherosclerotic plaque identification. As shown in human studies, the commercial USPIOs can be engulfed by macrophages *in vivo* with high efficiency, producing a detectable focal signal loss in MRI in proportion to the degree of atherosclerotic plaque inflammation. Thus, the USPIO-enhanced molecular MRI has been used to quantify the macrophage burden of plaque (Tang et al., 2008; Tang et al., 2009) and evaluate the rupture-prone plaques in patients (Morishige et al., 2010). These studies provided strong evidence of using nanoparticles to qualitatively and quantitatively evaluate plaque progression or vulnerability on the basis of nanoparticle deposition. Encouraged by the superb performance in clinical applications, the USPIOs with similar physicochemical properties are conjugated with various targeting moieties in animal models to expand their research studies on the identification of vulnerable components more precisely. An interesting example in this sense is a study by Wu et al. (2021), where they developed PP1 peptide-functionalized magnetic mesoporous silica nanoparticle (MSNP)-based bimodal imaging agents (PIMI) for the sensitive and specific assessment of macrophage burden in plaque in ApoE^−/−^ mice (Figure 1). PP1 was a peptide possessing targeting and antagonist ability to SR-AI scavenger receptors expressed on foamy macrophages (Segers et al., 2012) and could assist nanoparticles to highlight the plaque inflammation as reported in their preliminary study (Wang et al., 2019). As a result, PIMI exhibited a higher foamy macrophage deposition and remarkable contrast enhancement of plaque in *T*_*2*_-weighted and *T*_2_* mapping imaging compared with the MSNP without PP1 modification, suggesting that the active targeting strategies have great potential to discriminate the plaque inflammation with more sensitivity. On that basis, the research group further revealed that PIMI-enhanced MRI enabled precisely identifying the anatomic localization of foam macrophage-rich plaques in the aortic arteries, improving the characterization of plaque inflammatory activity, and monitoring the inflammatory process from an early to advanced stage of atherosclerosis. The results provided the convictive evidence for the feasibility of nanoparticle-assisted plaque risk stratification.

**FIGURE 2:**
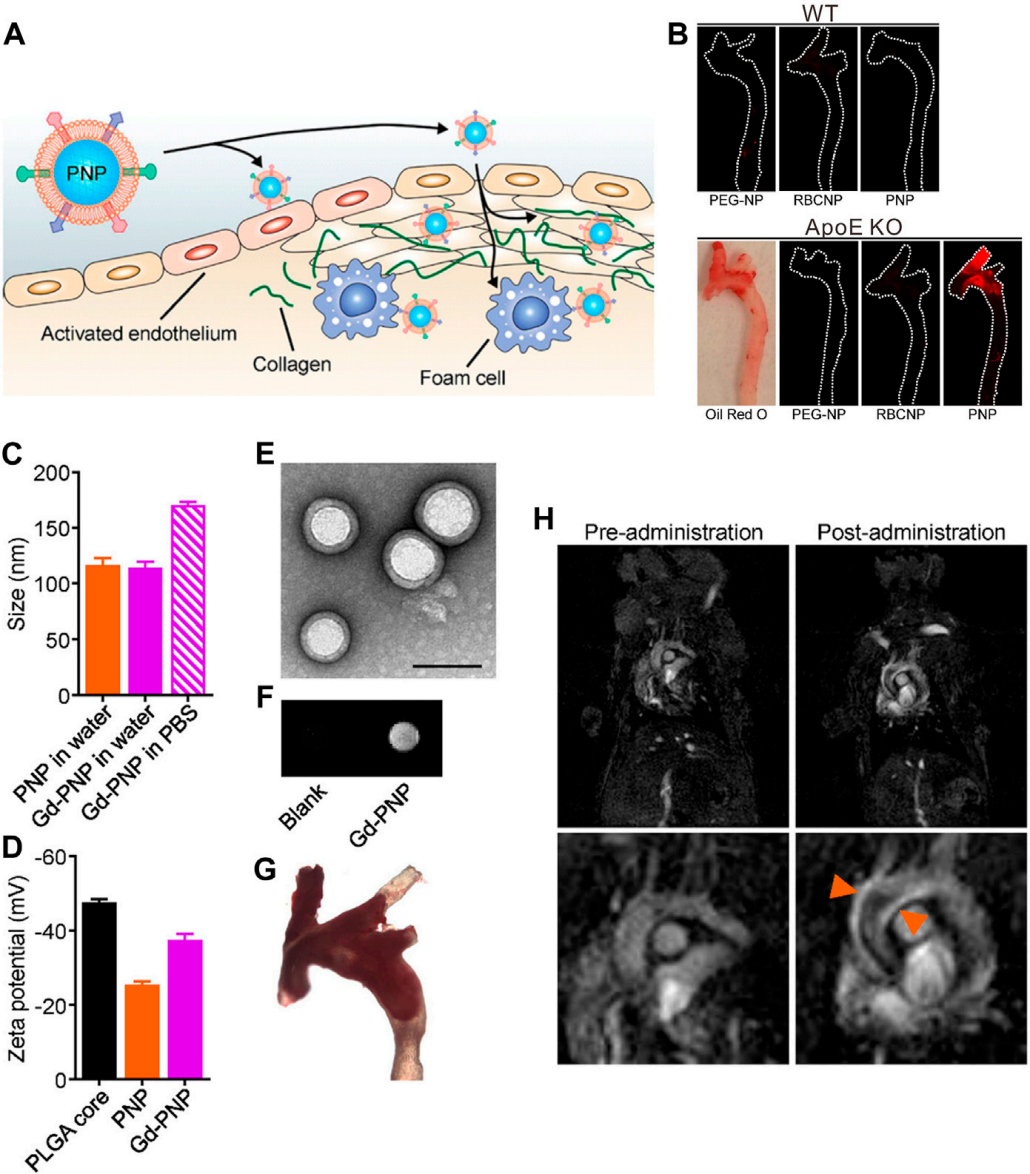
**(A)** Schematic illustration of PNPs targeting different components of atherosclerotic plaques. **(B)** Macroscopic fluorescent imaging of aortic arches from wild-type (WT) or ApoE KO mice fed on a high-fat western diet after intravenous administration with PEG-NPs, RBCNPs, or PNPs (white = physical outline, red = nanoparticle). Oil Red O staining was used to confirm the presence of plaque for ApoE KO mice (image is representative). **(C)** Particle size of PNPs and Gd-PNPs in water or PBS (*n* = 3, mean ± SD). **(D)** Surface zeta potential of bare PLGA cores, PNPs, and Gd-PNPs (*n* = 3, mean ± SD). **(E)** TEM image of Gd-PNPs (scale bar = 100 nm). **(F)** *In vitro T*_*1*_-weighted signal of Gd-PNPs. **(G)** Bright field image of aortic arch from ApoE^−/−^mice stained with Oil Red O confirmed the presence of atherosclerotic plaque. **(H)** *T*_*1*_-weighted MR images of ApoE^−/−^ mice before and 1 h after administration with Gd-PNPs (orange arrows = regions of positive contrast along the aortic arch) (Copyright permission from the American Chemical Society) (Wei et al., 2018).

Unlike *T*_*2*_ contrast agents producing “dark” MR signals, Gd-based *T*_*1*_ contrast agents usually generate “bright” MR signals to augment the contrast of target tissue, which are usually preferred in clinical applications in terms of clarity. However, limited by the resolution, identification of some the plaque microstructures such as the fibrous cap by MRI remains challenging. Owing to the critical role in the vulnerable plaque identification, several research groups have used Gd complex-containing nanoparticles to further assess the integrity of the fibrous cap. For instance, Ramirez-Carracedo et al. (2018) published an example of extracellular matrix metalloproteinase-inducer targeting imaging using gadolinium paramagnetic nanoparticles for the detection of MMP expression levels in plaque, showing reliable imaging of enzymatic changes related to the fragility of the fibrous cap (Ramirez-Carracedo et al., 2018). In another study, Wei et al. reported that gadolinium biomimetic nanoparticles could be used to visualize the risk of plaque and the vascular wall susceptible to plaque formation (Figure 2) (Wei et al., 2018). The gadolinium biomimetic nanoparticles with the particle size of ∼100 nm were constructed by coating the Gd-inserted platelet membrane on the prepared PLGA cores (Gd-PNPs). Due to the platelet membrane expressing a variety of surface markers, Gd-PNPs were observed to significantly enhance the contrast of plaque regions with endothelial dysfunction, fatty lesions, and collagen-contained fibrous cap *in vivo*, demonstrating their utilities of indicating the early plaque formation and unstable plaque transformation.

### Computed tomography

CT is considered a robust imaging technique for plaque identification in clinical trials. It can provide images to present the anatomic structure details of the coronary artery with short acquisition time, as well as enable post-processing of cross-sectional scans to render three-dimensional (3D) imaging with improved visual information. CT contrast relies on the intrinsic X-ray absorption of tissues, which means the tissues with higher density usually exhibit a higher X-ray attenuation [Hounsfield units (HU)]. From the plaque characterization perspective, CT reveals a superior performance in the context of diagnosing coronary calcium deposits, and the yielded calcium score has predictive value for future cardiovascular disease events (Hecht, 2015; Akyuz, 2020). Calcification can elevate mechanical wall stress and predispose the microfractures of plaque, having been regarded as a marker of accelerated atherosclerosis (Kataoka et al., 2012). Nevertheless, about three-quarters of all plaques are noncalcified. Because the X-ray attenuation of soft tissues is not sensitive enough, the vulnerability of such noncalcified plaques is difficult to be diagnosed from CT imaging. To highlight vulnerable components, CT contrast agents are usually used. Clinically approved CT contrast agents are small-molecular iodinated agents based on a tri-iodinated benzene ring with much higher X-ray attenuation than soft tissue (e.g., iohexol and iodixanol). However, the small molecular iodinated agents may be rapidly eliminated from the blood circulation in a matter of minutes after intravenous injection. In addition, CT suffers from low sensitivity to contrast agents, demanding high dosages of iodinated contrast agents to obtain clear CT images, which would cause potential renal toxicity and latent iodine hypersensitivity in specific patient populations. In this sense, nanoparticles allowing long-time circulation and enhanced delivery efficiency may be the new generations of CT contrast agents for high-performance plaque imaging.

Given that increased macrophage population in plaques has been regarded as a key feature of vulnerable plaques, Hyafil et al. designed the iodinated nanoparticulate contrast agent (N1177) for macrophage density imaging (Hyafil et al., 2007). N1177 exhibited an average diameter of 259 nm, which consisted of ethyl-3,5-bis(acetylamino)-2,4,6-triiodobenzoate in an amphiphilic tri-block copolymer. N1177 was deemed to be absorbed by macrophages in blood circulation and then infiltrated into plaque, allowing for imaging of the macrophage-rich region. By analyzing the results, they concluded that the atherosclerotic plaques with more than 20% of macrophage area could be identified in CT with an enhancement higher than 13.3 HU. On this basis, Ding et al. fabricated PEGylated lipids and stabilized the quantum dot-iodinated oil nanoemulsion platform for targeting visualization of plaque macrophages with combined CT and fluorescence imaging, which further confirmed the feasibility of evaluating the risk of plaque by imaging of macrophages (Ding et al., 2013).

**FIGURE 3:**
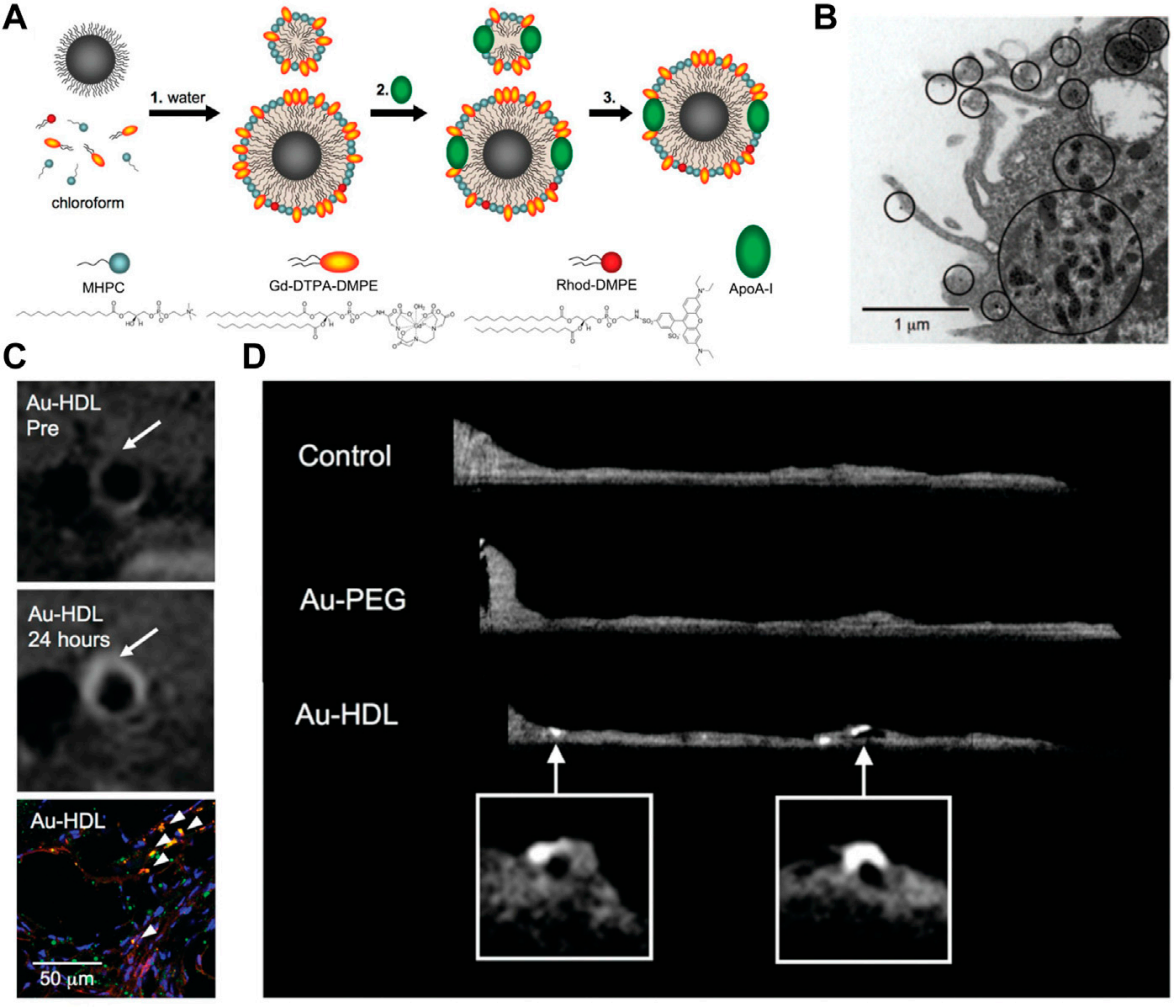
**(A)** Summary of the synthesis procedure of the agents. **(B)** TEM image of particle uptake. **(C)** *T*_*1*_-weighted MR images of the aorta of ApoE^−/−^ mice pre- and 24 h post-injection with Au-HDL, and the corresponding confocal microscopy images. **(D)** *Ex vivo* sagittal CT images of the aortas of mice injected with Au-HDL, Au-PEG, and saline (Copyright permission from the American Chemical Society) (Cormode et al., 2008).

**FIGURE 4:**
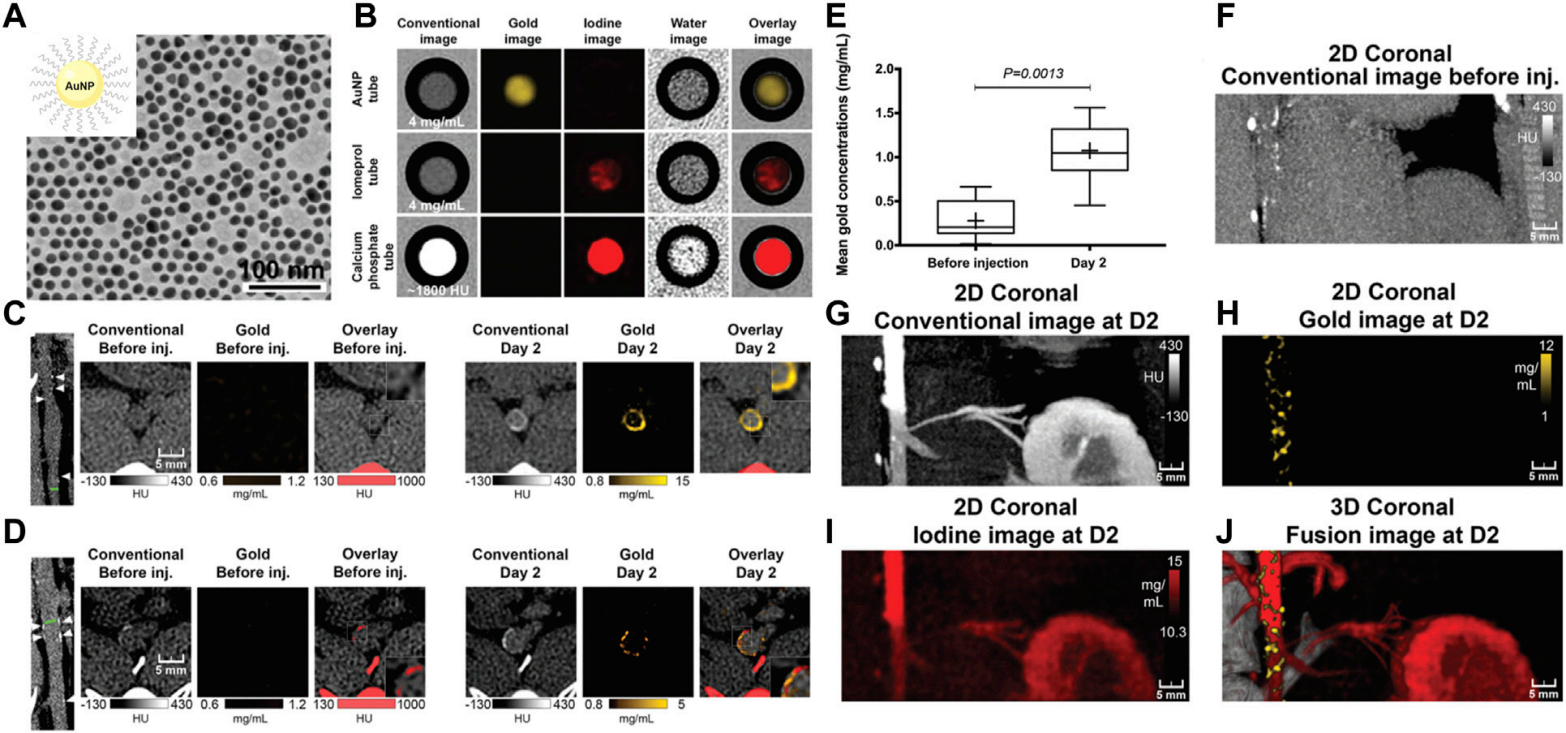
**(A)** TEM of gold nanoparticle. **(B)** *In vitro* photon-counting CT images of tubes containing Au nanoparticles (4 mg/ml), iomeprol (4 mg/ml), or calcium phosphate (1,800 HU). **(C)** Images show noncalcified plaque with strong circumferential enhancement and mean wall concentration of 4.5 mg/ml of Au nanoparticles. **(D)** Images show calcified plaque with strong enhancement within and around the calcified area and mean wall concentration of 2.74 mg/ml of Au nanoparticles. **(E)** Box-and-whisker plot shows quantitative analysis of mean Au concentration found in the whole atherosclerotic rabbit abdominal aortic wall before injection and 2 days after injection. **(F–J)** Photon-counting CT images of atherosclerotic rabbit aorta before and 2 days after injection of gold nanoparticles. 2D, two-dimensional. 3D, three-dimensional (Copyright permission from the Radiological Society of North America) (Si−Mohamed et al., 2021).

In addition to iodinated nanoparticles, inorganic nanoparticles consisting of high atomic number elements (e.g., Au, bismuth, tantalum, and ytterbium), which can provide improved X-ray attenuation than commercial iodine at CT energy ranges, received significant preclinical attention. Additionally, the density of the inorganic nanoparticles is much higher than that of iodine-based agents and can payload more contrast-generated elements. For example, Wang et al. (2016) reported an Au-based CT contrast agent with a cylinder shape (160 nm in length and 120 nm in width) could carry a payload of 7.7 × 10^5^ Au atoms per each nanoparticle. Of those, Au nanoparticles may be the most popular inorganic nanoparticles used in CT, since the elemental Au has almost three times greater X-ray attenuation per unit weight than that of iodine (Kim et al., 2007). In a landmark study in the field, Mulder et al. developed the HDL mimicking nanoparticles by incorporating Au nanoparticles for imaging macrophage expression in plaques in mice with CT (Figure 3) (Cormode et al., 2008). The prepared Au nanoparticle-based CT contrast agent was 9.6 nm in overall diameter, with superior attenuated X-rays at a rate of 1.5 times that of clinically used contrast agents (Omnipaque). Because HDL was the key protein to remove cholesterol from macrophages in plaques, more visible bright spots at the plaque lesion were observed in the group of Au-HDL nanoparticles compared with Au-PEG. Then, the same research group expanded upon the use of Au-HDL on multicolor CT for a more sophisticated characterization by distinguishing the spectral imaging of elemental Au (indicating the macrophage burden), iodine (indicating the stenosis of atherosclerotic plaques), and Ca_3_(PO4)_2_ (indicating the calcification) (Cormode et al., 2010). Multicolor CT imaging is a powerful technology developed in the past decade that allows to simultaneously identify several accumulations of contrast materials by separating the transmitted X-rays into multiple energy bins (de Weert et al., 2006; Shinohara et al., 2008; Wehrse et al., 2021). Distinguishing multiple contrast material types in CT signifies the opportunity for more precise delineation between vulnerable components phagocytosed by nanoparticles with different elements, or between nanoparticles deposition and high attenuating tissue. An impressive study by Douek et al. revealed the feasibility of combination of Au nanoparticles and the most advanced multicolor CT technology (photon-counting CT) to characterize the macrophage burden within calcified atherosclerotic plaques (Figure 4) (Si−Mohamed et al., 2021). Au nanoparticles with a mean hydrodynamic diameter of 18 nm that could be engulfed by macrophage *in vivo* were used as the CT contrast agents. Multicolor CT images revealed the distribution of Au nanoparticles in the aortic wall was clearly separated from the calcified region at the area of plaque. Transmission electron microscopy and inductively coupled plasma optical emission spectrometry analyses confirmed the results of Au-specific imaging findings.

### Position emission tomography and single photon emission computed tomography imaging

PET and SPECT are the nuclear imaging techniques that require radiotracers to quantitatively detect molecular targets within picomolar concentrations, which have been applied in recent years to quantify pathological processes occurring within the arterial system (Tarkin et al., 2014; Tawakol et al., 2017). ^18^F-fluorodeoxyglucose (^18^F-FDG) is the most commonly used radiotracer in clinic. Because ^18^F-FDG can easily be taken up by the glucose transporter, it has been regarded as the metabolic marker to indicate the increased metabolic activity of macrophages and vascular inflammation (Tawakol et al., 2006). In addition to that, ^68^Ga-DOTATATE and ^68^Ga-PENTAXIFOR have also been recently introduced as the PET imaging agents in patients for their specificity to macrophages and CXCR4 receptors, respectively (Thackeray et al., 2015; Bozkurt et al., 2017). However, several intrinsic limitations of PET/SPECT remain challenges for precise characterization of vulnerability of atherosclerotic plaque. The major disadvantage of this imaging modality is the poor spatial resolution, which makes the quantification of arterial activity to be influenced by signal “spill out” (signal loss to surrounding tissue or signal) or “spill in” (signal added from neighboring structures) (Tarkin et al., 2014). Other disadvantages are the non-specificity and rapid clearance of radiotracers, rendering imaging of the coronary arteries particularly challenging due to avidly nonspecific uptake (Lobatto et al., 2011). In addition to that, PET or SPECT cannot provide anatomical information, which requires them to combine with CT or MRI for the anatomical reference (Tarkin et al., 2014; Cheng et al., 2015). Therefore, for the sophisticated characterization of atherosclerotic plaques, increasing plaque targeting ability and improving the signal-to-noise ratio are of intense significance for radiotracers.

**FIGURE 5:**
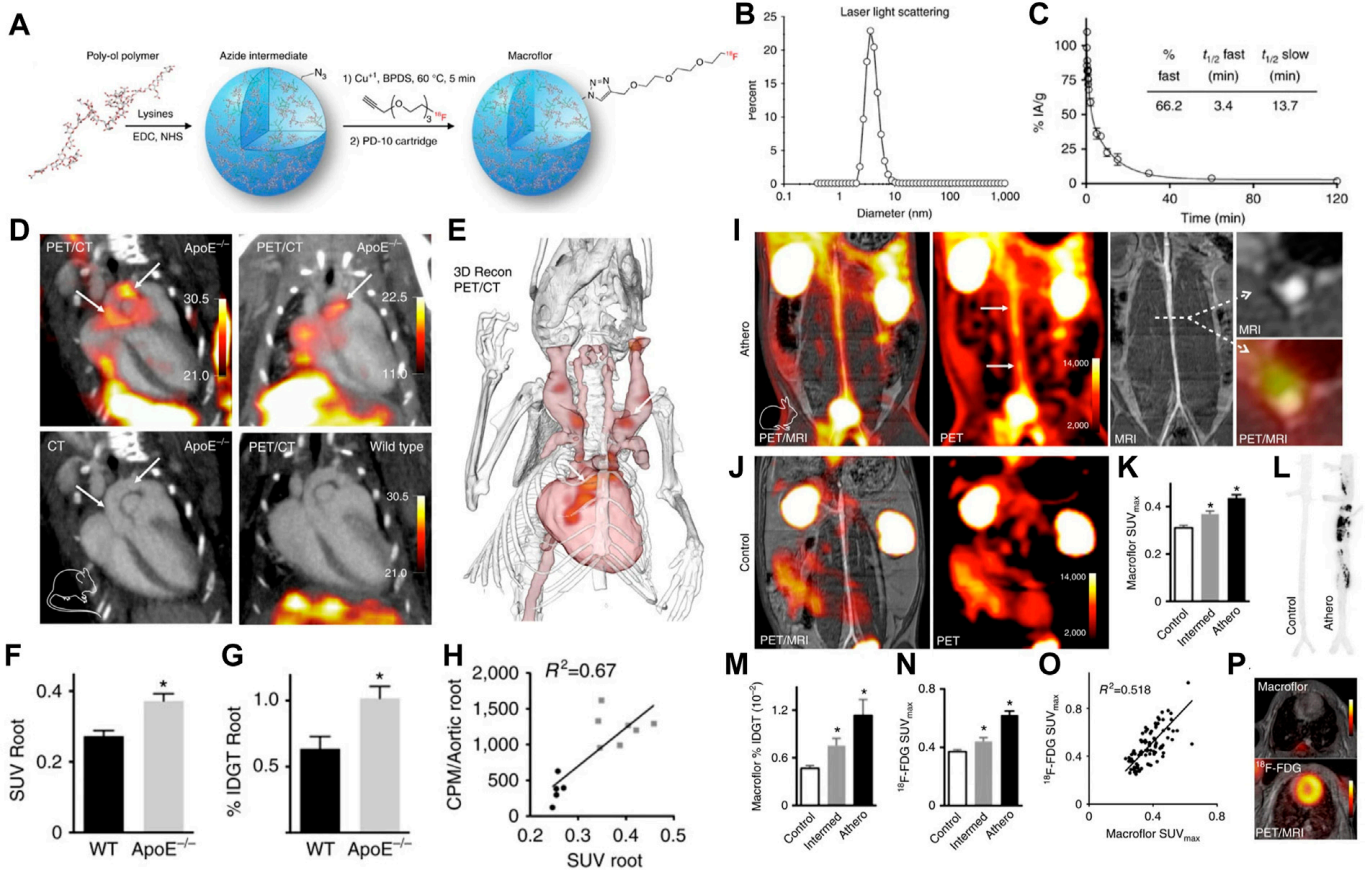
**(A)** Synthesis and radiolabeling scheme of 18F-Macroflor. PET/MRI on **(B)** dynamic light scattering measurement of purified nanoparticle. **(C)** Size-exclusion chromatogram of purified labeled and unlabeled nanoparticles. **(D)** Representative PET/CT images of several experiments in ApoE/and wild-type control mice after IV Macroflor injection. PET scale bar is in kBq/ccn ¼ 14. **(E)** Three-dimensional rendering derived from PET/CT in the ApoE/mouse shows the PET signal in red (arrows). **(F)** *In vivo* standard uptake values (SUVs) for aortic roots of wild-type and ApoE/mice (*n* ¼ 5–7 per group, unpaired *t*-test). **(G)** *Ex vivo* gamma count reports percent injected dose per gram aortic tissue. **(H)** Correlation of (C, D) for individual wild-type (black) and ApoE/mice (gray), counts per minute (CPM). **(I)** PET/MR images obtained in rabbits with atherosclerosis and **(J)** control rabbits. **(K)** Standard uptake values (SUVs) in the infrarenal aorta after Macroflor injection in control rabbits, rabbits with intermediate and full aortic balloon infrarenal aorta denudation. **(L)** Autoradiography of the abdominal aorta in a control rabbit and a rabbit with atherosclerosis after Macroflor injection, representative images of 12 autoradiography exposures (*n* ¼ 4 per group) **(M)** *Ex vivo* gamma counting and **(N)** aortic SUV in infrarenal aorta of the same rabbits 2 days before Macroflor injection. **(O)** Correlation of 18F-FDG with Macroflor *in vivo* PET signal. **(P)** Cardiac PET images with respective agents. (Copyright permission from Springer Nature) (Keliher et al., 2017).

To overcome these aforementioned limitations, nanoparticles that can be labeled with radionuclides or targeting elements in a controlled manner have been proposed to serve as valuable tracers for PET/SPECT. For example, Beldman et al. (2017) used ^89^Zr-labeled hyaluronan nanoparticles (^89^Zr-HA NPs) for combined PET/MR imaging, in which the ^89^Zr-HA NPs showed enhanced accumulation in atherosclerotic macrophages, proving a feasible strategy to imaging the local inflammatory activity. In another study, an important vulnerable feature of necrotic lipid core within plaques was successfully visualized on SPECT/CT imaging by using the ^99^mTc-labeled annexin V-modified hybrid gold nanoparticles (^99^mTc-GNPs-Annexin V) as the radiotracers (Li, et al., 2016). In an impressive study of nanoparticle application in PET plaque imaging, Keliher designed ^18^F-labeled polyglucose nanoparticles (^18^F-Macrins) to visualize inflammation in atherosclerotic plaque and ischemic myocardium in mouse and rabbit models. Owing to the Macroflor’s high avidity for macrophages, imaging patterns of ^18^F-Macrins can be used to detect the changes in macrophage population size and indicate the increasing or resolving inflammation with plaque (Figure 5) (Keliher et al., 2017). Moreover, in order to optimize the pharmacokinetics profile of ^18^F-Macrins to match with the rapid radioactive half-life of ^18^F (^18^F, *T*_*1/2*_ = 110 min), the authors shrank the size of ^18^F-Macrins to a mean particle diameter of 5.0 ± 0.4 nm. The markedly smaller size facilitated the rapid pharmacokinetics profile and favorable macrophage uptake of ^18^F-Macrins, providing new opportunities for ^18^F in nanomedicine.

**FIGURE 6:**
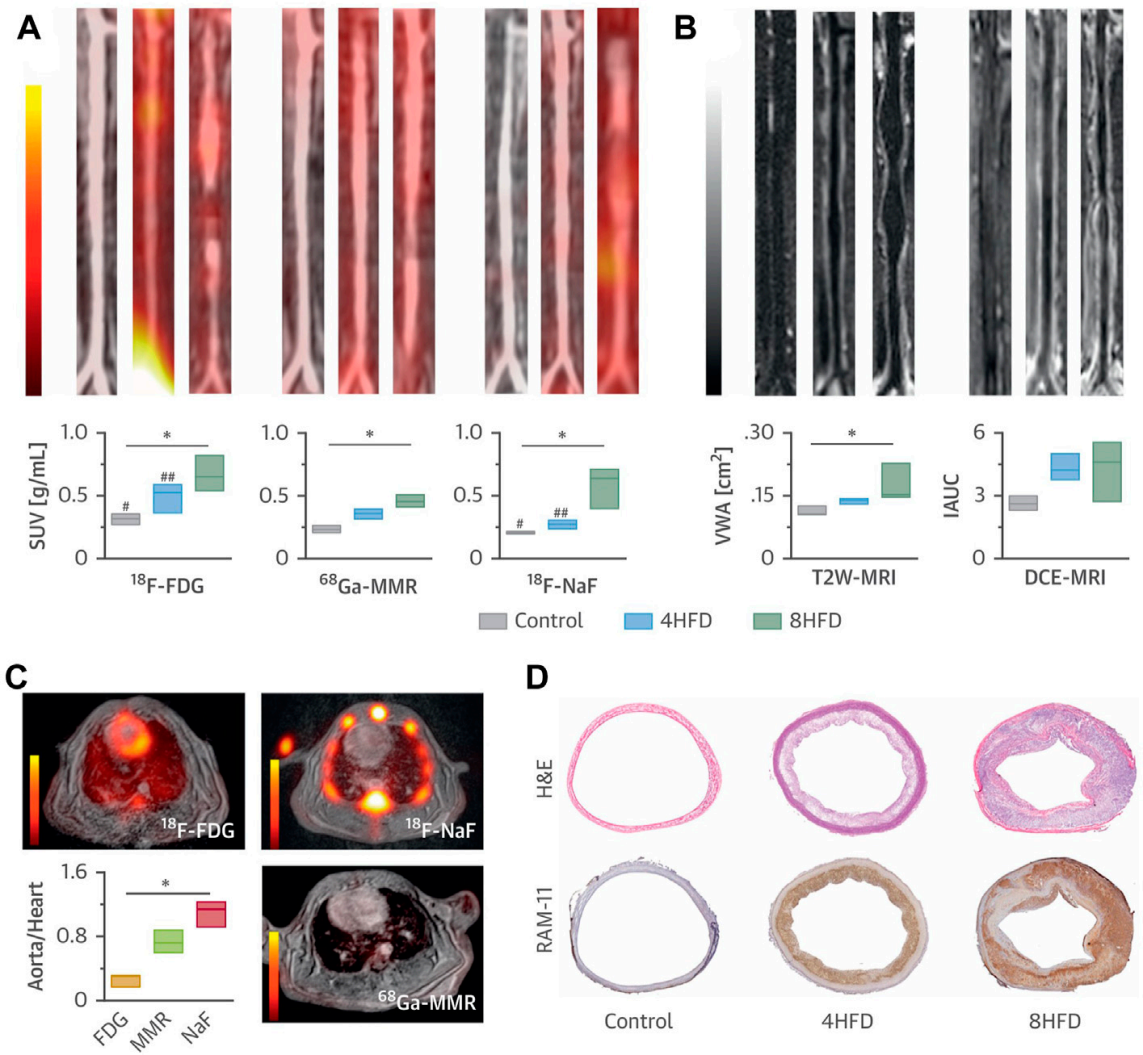
**(A)** Representative coronal aortic fused PET/MR images for ^18^F-FDG (3 h) (left), ^68^Ga-MMR (2 h) (middle) and ^18^F-NaF (1.5 h) (right), and **(B)** representative *T*_*2*_W-MRI (left) and DCE-MRI (right) images from healthy and atherosclerotic rabbits (on high-fat diet for 4 months or 8 months, *n* ≥ 3 per group). **(C)** Cardiac PET/MR images of the respective tracers and associated aorta-to-heart ratios in rabbits with atherosclerosis (8 HFD). **(D)** Aortic sections taken from healthy control subjects and atherosclerotic rabbits (4 HFD or 8 HFD) and stained with H&E and RAM-11 (macrophages). *p < 0.05; ^18^ *F*-FDG versus ^18^ *F*-NaF: ^#^ *p* < 0.05; ^##^ *p* < 0.01 (Copyright permission from Elsevier) (Senders et al., 2018).

In another study, Medina et al. synthesized several kinds of radionuclide-labeled nano-scaled antibodies (termed nanobody: ^64^Cu-VCAM-1, ^64^Cu-LOX-1, ^64^Cu-MMR, and ^68^Ga-MMR), and used them as radiotracers for multiparametric PET/MRI imaging (Figure 6) (Senders et al., 2018). The purpose of the study was to investigate whether the combination of molecular PET, *T*_*2*_-weighted-MRI, and DCE-MRI can be used to comprehensively evaluate atherosclerosis progression. Specifically, this multiparametric imaging approach was executed as follows: first, ^64^Cu-VCAM-1, ^64^Cu-LOX-1, and ^64^Cu-MMR were used to characterize the endothelial dysfunction and macrophage burden of plaque and inflammatory activity; then ^68^Ga-MMR was integrated with *T*_*2*_-weighted-MRI and DCE-MRI on a clinical PET/MRI system to simultaneously investigate vessel wall morphology and atherosclerotic plaque activity; meanwhile, ^18^F-FDG-PET and ^18^F-NaF-PET were carried out to study atherosclerosis progression. In this study, the nanobodies were shrunk from 150 KD to 10∼5 KD. Owing to the rapid pharmacokinetics profile and high affinity to plaques, these nanobodies tremendously facilitated the imaging of the vessel wall and the highlight of vulnerable components, which could be used for comprehensive estimation of overall plaque activity or precise analysis of the specific vascular segment. In this regard, this study provided a valuable example of nanoparticles to be customized as clinically available contrast agents to facile the noninvasively monitoring of atherosclerosis progression.

### Ultrasound imaging

US is a mature clinical imaging technology for vascular diseases. Due to their limitations of insufficient penetration, low spatial resolution, and insensitivity, contrast agents such as microbubbles (MBs) or other acoustically active micro- or nanobubbles (NBs) are usually required (Alphandéry, 2022). MBs or NBs are often filled with gases in the internal core; thus, they can create differences between the location sites and the surrounding tissues in echogenicity patterns, increasing ultrasound resolution and allowing visualization of targeted plaque composition. The most unique advantages of US are the low-cost and fast imaging in real-time, which means that rapid molecular imaging of atherosclerosis with portable and relatively inexpensive imaging technology may be possible in future. Furthermore, from a research perspective, molecular US is an ideal tool for rapid and non-invasive evaluation of the process of atherosclerotic plaque, especially in the early stages.

**FIGURE 7:**
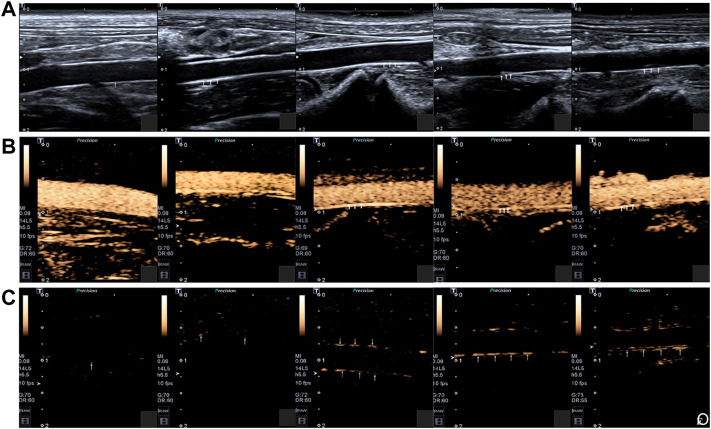
**(A)** Two-dimensional ultrasonography images of the abdominal aorta of rabbits in different groups. **(B)** SonoVue ultrasonic contrast images of the abdominal aorta of rabbits in different groups. **(C)** ICAM-1-targeted nano ultrasonic contrast images of the abdominal aorta of experiment rabbits in different groups. Five rabbits in each group: control group; week-4 group; week-8 group; week-12 group; week-16 group. The intima-media membrane and the plaque on the wall were indicated by the arrows (Copyright permission from Springer Nature) (Li et al., 2021).

**FIGURE 8:**
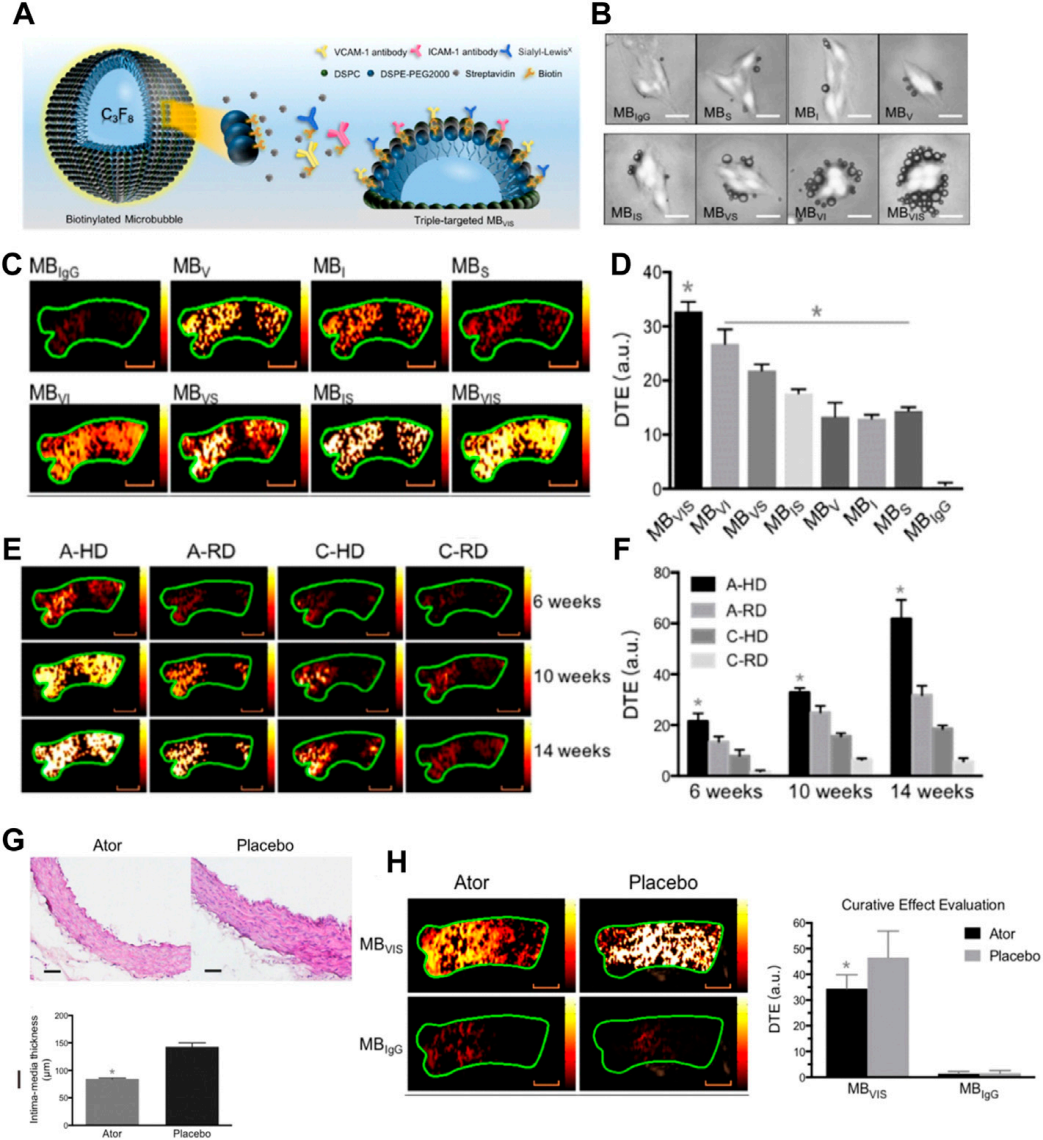
**(A)** Diagram of targeted MB_VIS_. **(B)** Representative bright-field micrographs of targeted MBs and MB_IgG_ bound to stimulated bEend.3 cells (40 ng/ml TNF-α; scale bar = 10 μm). **(C)** Representative color-coded ultrasound images after injection of various kinds of MBs at the 10-week feeding time, and **(D)** the quantitative analysis of ultrasound signal intensities. **(E)** Representative color-coded images from four groups after injection of MB_VIS_, and **(F)** the quantitative analysis of ultrasound signal intensities. Scale bar = 1 mm. **(G)** Representative H&E images and quantitative analysis of intima-media thickness of ascending aorta sections from ApoE^−/−^ mice-fed atorvastatin or placebo in their daily hypercholesterolemic diets for 8 weeks. Scale bar = 50 μm. *p < 0.05. **(H)** Representative color-coded images of ascending aorta and quantitative analysis of signal intensities generated from adherent MB_VIS_ or MB_IgG_. Scale bar = 1 mm *p < 0.05 (Copyright permission from the Ivyspring International Publisher) (Fei et al., 2018).

For example, in a study of the atherosclerotic non-human primates’ model, MBs targeting P-selectin (MBP) and VCAM-1 (MBV) had been cobducted to evaluate the earliest stages of endothelial inflammatory changes prior to carotid intimal thickening (Chadderdon et al., 2014). In another study, Yang et al. (2016) sought to explore the feasibility of the ICAM-1 antibodies decorated with NBs (nano-ultrasonic contrast agent) to evaluate the degree of inflammatory injury at different stages in the atherosclerotic rabbits (Figure 7) (Li et al., 2021). They found that ICAM-1-targeted ultrasonic nanobubbles could specifically interact with the endothelium with upregulated expression of ICAM-1, enabling specific imaging of atherosclerotic inflammation. Histological verification showed that the imaging intensity and imaging time window of ICAM-1-targeted ultrasonic nanobubbles had a stronger positive linear correlation with the severity of inflammation than that of MBs. The author supposed the possible reason may be that the NBs could be quickly endocytosed into cells after adhering to the targeted cells, while the endocytosis of MBs was much more difficult because of their large size. Thus, the MBs in the targeted sites would fade rapidly. In addition to that, the author mentioned that the inadequate binding rate of ICAM-1 antibody to NBs might affect the targeting and imaging efficiency in large arterial vessels that had high blood shear stresses. To address this issue, scholars had attempted to utilize the external force [e.g., acoustic radiation force (Liu et al., 2012) and magnetic field force (Wu et al., 2011)] or enhance the amount of targeting motifs to improve the targeting ability of ultrasonic contrast agents (Weller et al., 2005). Recently, Zheng et al. (2019) developed triple-targeted microbubbles (MBs) (VCAM-1/ICAM-1/P-selectin-targeted MB_VIS_) to improve the targeted MB adhesion efficiency at the early stage when there was no obvious plaque in the lumen (Figure 8) (Fei et al., 2018). The polymeric sLex ligand was used to mimic the migration behavior of leukocytes during the development of plaque, while the antibodies of VCAM-1 and ICAM-1 were used to target VCAM-1, ICAM-1, and P-selectin, resulting in a nearly 3-fold higher binding number than the dual-/single-targeted MBs in the ApoE^−/−^ mice.

### Optical imaging

Non-invasive optical imaging technologies, such as optical fluorescence and bioluminescence imaging, have experienced an incremental improvement in preclinical biomedical imaging due to their high spatial resolution, outstanding sensitivity, fast acquisition, and cost-effectiveness. More importantly, their multispectral potential allows simultaneous visualization of multiple molecular epitopes, providing a great opportunity for characterization of several vulnerable features with high throughput. Generally, optical contrast is generated by detecting fluorophore emission spectra, fluorescence lifetime, molecular resonances, or photoacoustic waves. However, due to the limited penetration depth of light and the undesirable overlap with autofluorescence of plaque tissues, the non-invasive optical imaging of atherosclerotic plaque, especially located in deep organs such as the aorta or coronary artery, has been seriously confined. In this context, optical contrast agents that enable enhanced tissue penetration and reduced tissue autofluorescence background are of intense interest for the sophisticated characterization of atherosclerosis.

**FIGURE 9:**
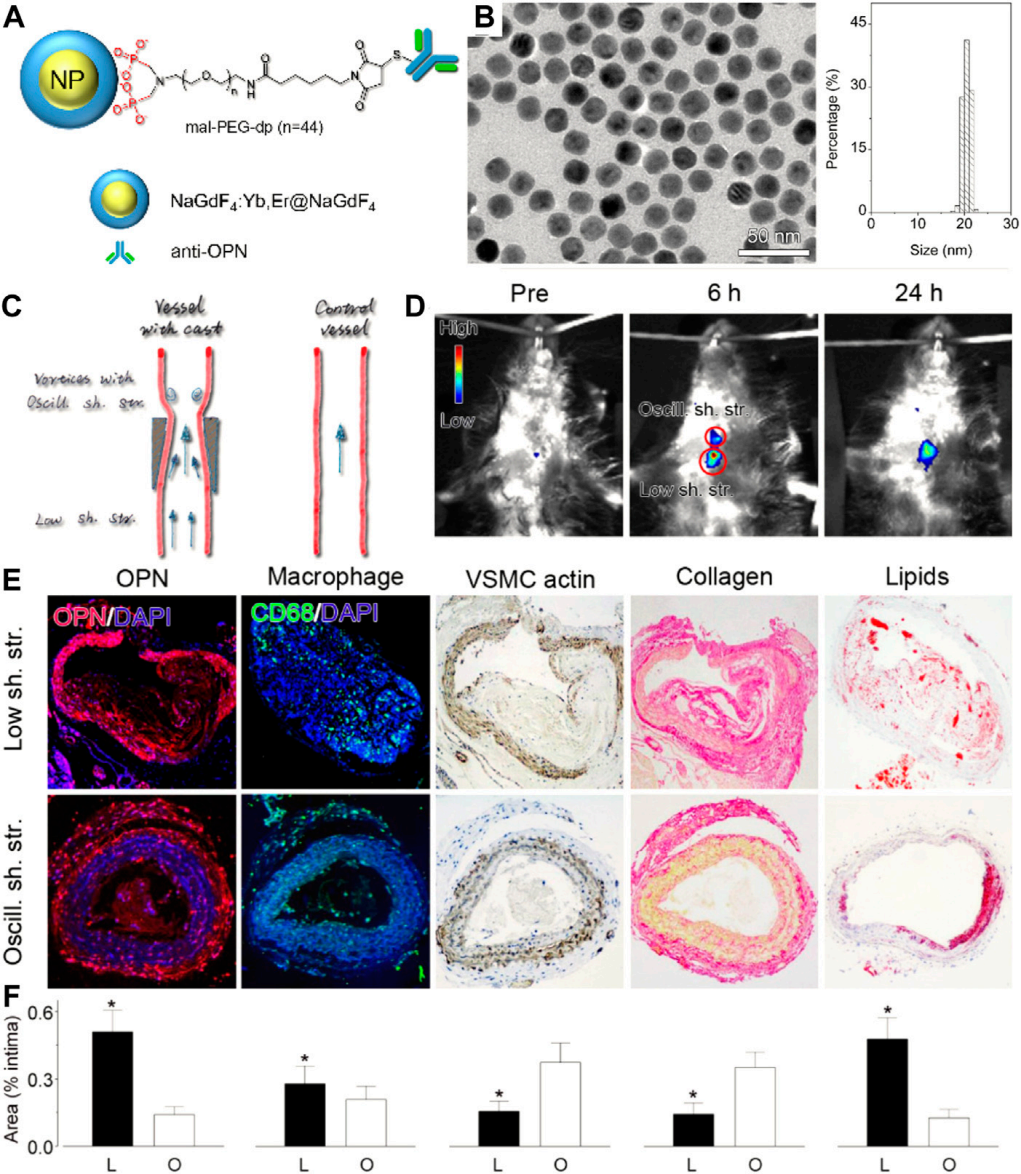
**(A)** Schematic diagram and **(B)** TEM images and size histograms of the UCNP-anti-OPN probe. **(C)** Schematic diagram for showing the varied stress-induced plagues in ApoE^−/−^ mice. **(D)** *In vivo* upconversion luminescent images captured before and at different time points after intravenous injection of the UCNP-anti-OPN probe. **(E,F)** Histological analyses and quantified data of the different plaque regions upon various staining (*p < 0.05) (Copyright permission from the American Chemical Society) (Qiao et al., 2017).

Near-infrared fluorescent (NIF) probes, with emission peaks in the range of near-infrared windows, can provide higher signal-to-background ratios than visible probes (Hu et al., 2018). NIF nanoparticles can be obtained from loading NIR dyes on/in nanoparticles, or fabricating synthetic upconversion nanoparticles, carbon-based nanoparticles, or semiconductor quantum dots (QDs), having been broadly explored to probe vulnerable markers at the molecular level. For instance, specific NIF imaging of macrophages in vulnerable plaques was demonstrated based on indocyanine green-labeled phosphatidylserine liposomes with 180 nm (Narita et al., 2019). Cy5.5- or Cy7-decorated glycol chitosan nanoparticles had been used as MMR probes to specifically image thin-cap fibroatheroma within high-risk plaques (Kim et al., 2016). In a representative study of upconversion nanoparticle-based vulnerable plaque imaging, Qiao et al. (2017) reported that NIF NaGdF_4_:Yb,Er@NaGdF_4_ upconversion nanoparticles (UCNPs) conjugated with antibodies against foamy macrophage specific receptor osteopontin (UCNP-anti-OPN) were used to fluorescently image pro-inflammatory foamy macrophages (Figure 9). The prepared UCNP-anti-OPN is electroneutral and highly monodispersed with an average size of 18.3 nm. Based on the fact that foamy macrophages are richer in vulnerable plaques than those in stable plaques, the UCNP-anti-OPN can distinguish rupture-prone plaques from those of stable ones by probing the expression of osteopontin, demonstrating the NIF nanoparticles’ potential and feasibility in fluorescence imaging of atherosclerosis. Remarkably, the NIR-II optical imaging (NIR-II, 1,000–1,700 nm), developed in the past decade, has shown great promise to provide biological structures and process information of vessels with a higher signal-to-background ratio, deeper penetration ability, and higher resolution, because tissues in the longwave region become partially transparent as their extinction coefficient are minimized (Wang et al., 2021). For example, Zhang et al. (2021) developed a novel human single-chain variable fragment (scFv) antibody with functionalized NaNdF_4_@NaGdF_4_ nanoparticles to quantify the oxidation-specific epitope (OSE) level in atherosclerotic plaques. The OSE level was inversely associated with the incidence of carotid atherosclerosis. Taking advantage of high spatiotemporal resolution and an outstanding signal-to-background ratio of NIR-II optical imaging, the OSE level within plaques was correctly identified and the degree of vulnerability was indirectly evaluated from NIR-II fluorescence imaging within a short scanning time.

**FIGURE 10:**
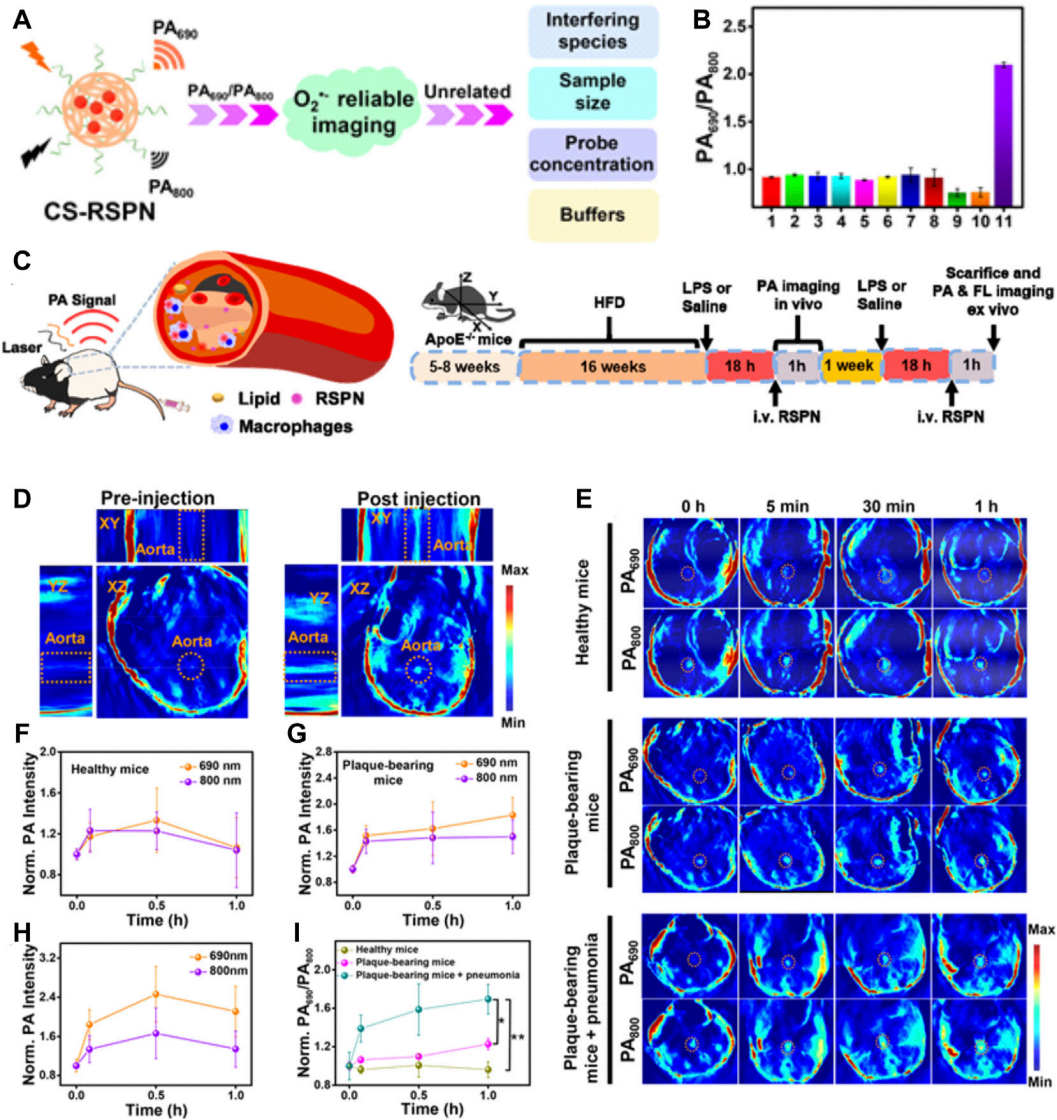
**(A)** Schematic illustration of CS-RSPN for reliable ratiometric photoacoustic imaging of O_2_^−^. **(B)** Selective response: PA690/PA800 ratios of RSPN. **(C)** Scheme of RSPN for photoacoustic imaging of atherosclerotic plaques and administration procedure (*n* = 3). **(D)** 3D PA image of plaque-bearing mice before and after injection of RSPN. Excitation: 690 nm. Aorta regions are depicted by dotted circles. **(E)** PA images after injection of RSPN of healthy mice, plaque-bearing mice, and plaque-bearing mice complicated with pneumonia. Data are shown as mean ± SD (*n* = 3). Aorta regions are depicted by dotted circles. **(F–I)** Normalized PA690 and PA800 of healthy mice, plaque-bearing mice, and plaque-bearing mice complicated with pneumonia (two-tailed Student’s *t* test, *p < 0.05, **p < 0.01). (Copyright permission from the American Chemical Society) (Ma et al., 2021).

In addition to fluorescence imaging, another recently emerged optical imaging modality showing great potential for accurate diagnosis of vulnerable plaque is photoacoustic imaging (PAI). PAI can transform the optical energy into acoustic signals; therefore, it is a highly complementary modality possessing superiorities in the imaging depth of ultrasonography and the sensitivity of optical imaging (Steinberg et al., 2019). Generally, both the endogenous tissues and exogenous agents with light-absorbing ability to cause thermal expansion and generate ultrasonic waves under pulsed laser irradiation can be used for PAI. On that basis, NIF dye-labeled nanoparticles, metallic or semiconducting nanoparticles, and NIF organic nanoparticles are proposed to be exploited as molecular PA contrast agents for targeting imaging the atherosclerosis biomarkers. For instance, Ge et al. (2020) showed the feasibility of screening the critical ingredients of atherosclerotic plaque at the molecule level in mice by using the anti-osteopontin antibody (OPN Ab) and NIR dyes with an ICG-functionalized nanosized platform (OPN Ab/Ti_3_C_2_/ICG) as the PA contrast agents. In another study, Gifani developed a SWNT-based ultraselective imaging methodology to precisely and specifically identify vulnerable plaque by highlighting the regions severely infiltrated by Ly-6Chi monocytes and foamy macrophages (Gifani et al., 2021). The results of PAI are highly in parallel with the results of high-dimensional flow cytometry studies, confirming the high selectivity of this SWNT-based PAI strategy. Due to the predominant sensitivity and signal-to-background ratio, nanoparticle-enhanced PAI has the potential to explore more varieties of vulnerable plaques’ indicators which may be easily degradable or have a shorter half-life. For example, Ma et al. (2021) represented a fascinating design of the ratiometric semiconducting polymer nanoparticles (RSPNPs) to dynamically assess the O^2−^ levels within plaques (Figure 10). O^2•−^ was the critical substrate for the oxidation of LDL and the synthesis of inflammatory enzymes, having been regarded as a critical indicator responsible for the plaque vulnerability. However, it was difficult to be detected by the conventional clinical imaging technique due to its extremely short half-life and low local concentration. In this study, the authors used the O^2•−^-responsive molecule (ORM) to capture intraplaque O^2•–^ to offer robust “turn on” absorption at 690 nm; while the insensitive semiconducting polymer molecule (OIM) with a signal at 800 nm was served as an internal photoacoustic reference. By monitoring the changes in photoacoustic signals at 690 nm and 800 nm (PA690/PA800), the O^2•–^ level within plaques could be identified to assess the rupture risk of atherosclerotic plaques.

### Multimodality imaging

**FIGURE 11:**
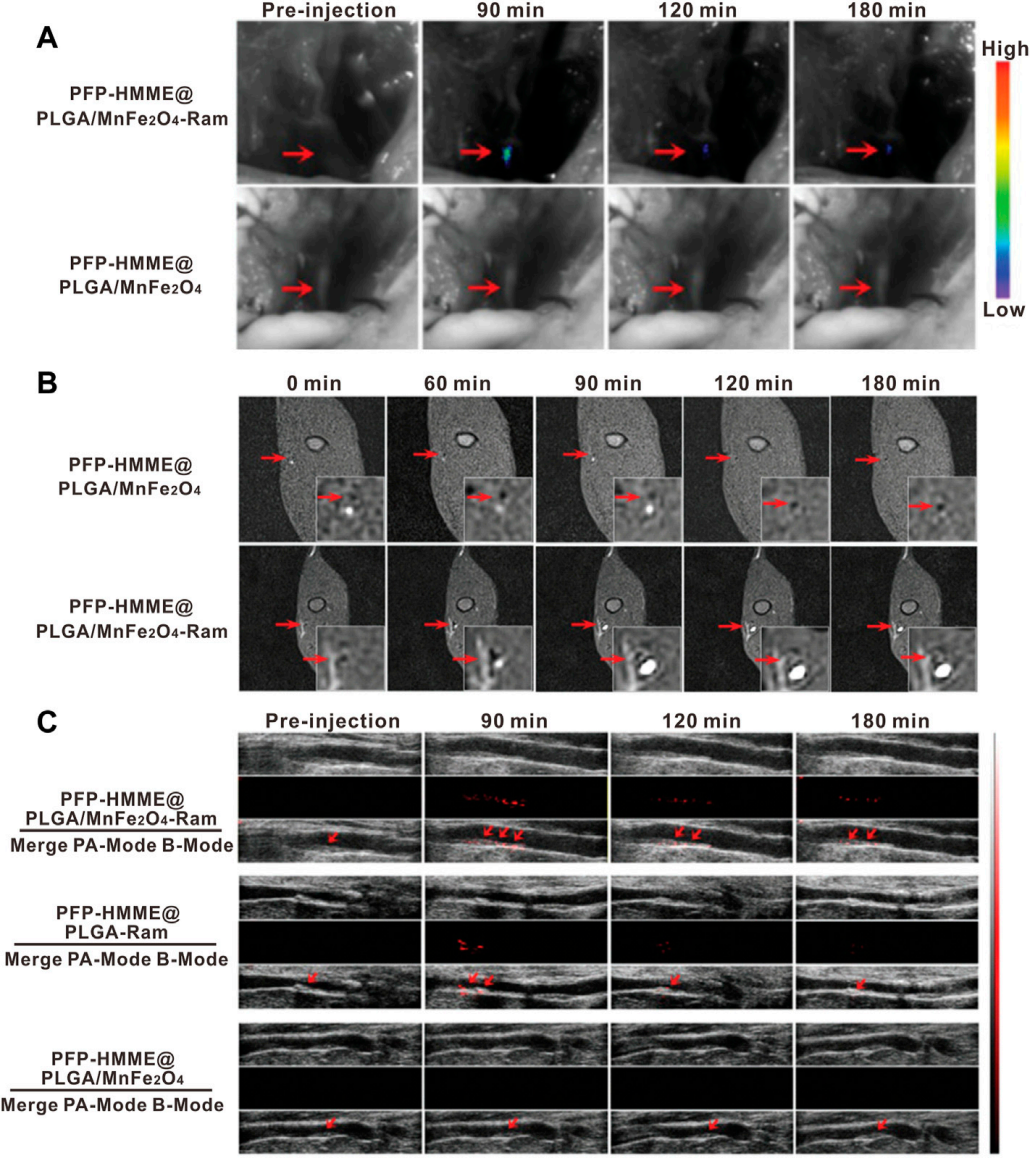
*In vivo* neovessel-targeting behavior of PFP-HMME@PLGA/MnFe_2_O_4_-Ram NPs. The bright-field and **(A)** near-infrared fluorescence images, **(B)** MRI *T*_*1*_ images, and **(C)** US of femoral plaque-bearing rabbits after intravenous injection of targeted NPs and nontargeted NPs at different time points (Copyright permission from Wiley-VCH GmbH) (Yao et al., 2021).

The multicomponent nature of nanoparticles offers a diverse platform to hybrid two or more imaging modalities within the same nanoparticle to achieve synergistic advantages over any single modality alone (Louie, 2010). Given the high heterogeneity and complexity of plaque composition, multifactorial pathophysiology of atherosclerosis, multimodality imaging that can provide complementary information from different angles has brought new perceptions to the fields of high-risk feature evaluation and the risk plaque identification. Nowadays, advances in multimodality techniques and imaging contrast agents have generated great innovations. For instance, clinically used PET/CT (Kwiecinski et al., 2021) or PET/MR (Aizaz et al., 2020) imaging is the typical application of combined imaging profile to yield molecular, functional, and anatomical information together for a better characterization of plaques. Motivated by the rapid development of materials science, multimodal nanoparticles for dual-, tri-, and even multi-modal combined imaging of atherosclerosis are undergoing intensive exploration. For example, Wang et al. (2019) constructed an optical/MR dual-modality imaging probe (anti-MARCO UCNPs) by conjugating the polyclonal collagenous structure (MARCO) antibody to the surface of the prepared upconversion nanoparticles *via* the condensation reaction. As the drawback of optical imaging was the limited tissue penetrance and the lack of anatomical information and the MRI not being sensitive enough, the optical/MR dual-modality imaging would generate an efficient imaging with high sensitivity and high spatial resolution. The imaging results demonstrated the superiority of combined morphological and molecular imaging in visualization of M1 phenotype macrophages and identification of vulnerable plaques. Yao et al. (2021) developed the multimodal (MRI/PA/US) nanoparticles by encapsulated manganese ferrite (MnFe_2_O_4_), hematoporphyrin monomethyl ether (HMME), and perfluoropentane (PFP) into the polylactic acid–glycolic acid (PLGA) shells (Figure 11). After being functionalized with the anti-VEGFR-2 antibody, the prepared PHPMR NPs could bind to plaque neovascularization and provide multimodal monitoring in real time.

### Treatment monitoring and imaging-mediated therapeutics

**FIGURE 12:**
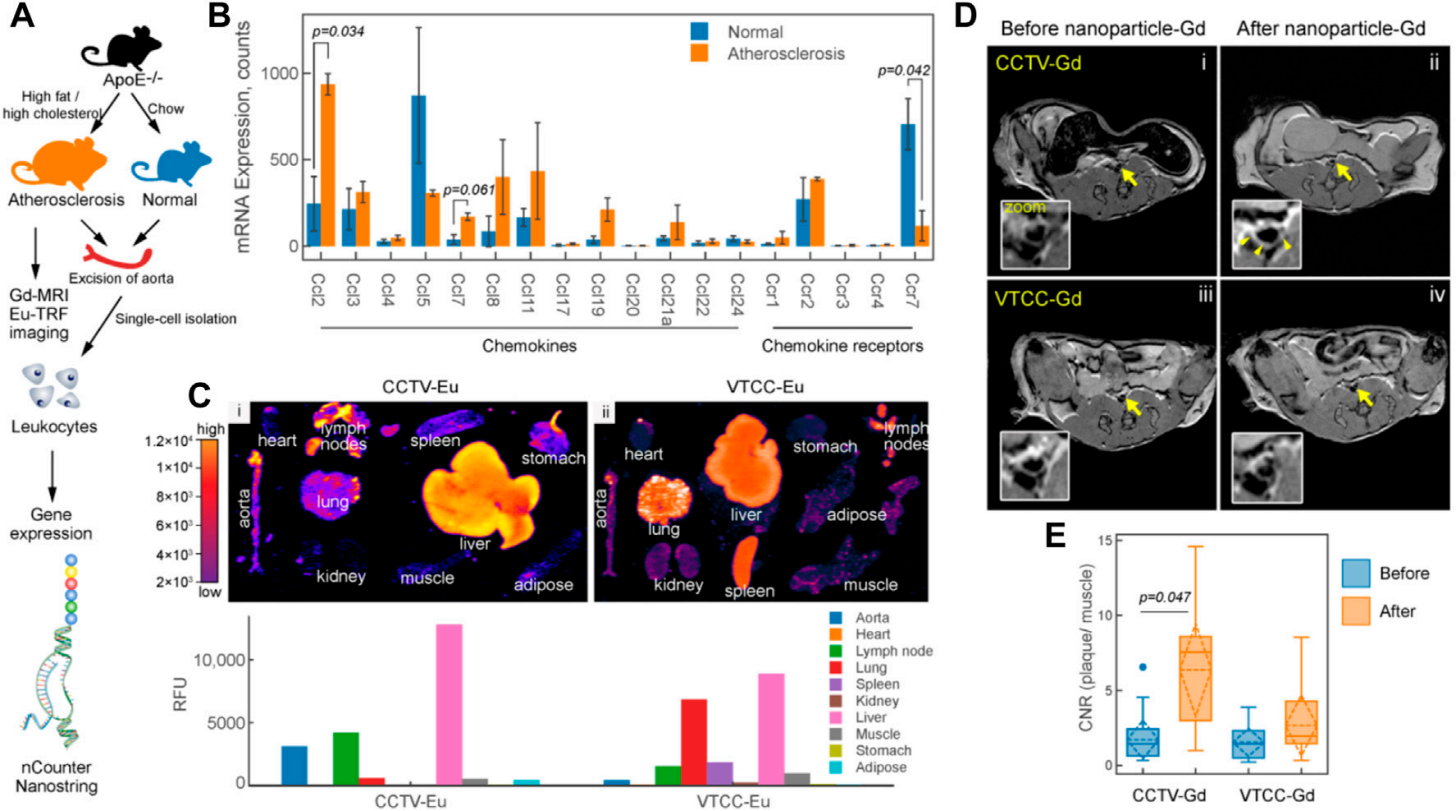
CCR2/CCL2 is highly expressed in atherosclerosis and a target for CCTV contrast agents. **(A)** Experimental workflow and generation of the atherosclerosis model. **(B)** nCounter Nanostring assays enabled quantification of mRNA expression of major chemokines and their receptors in leukocytes isolated from aortas of normal and atherosclerotic mice. **(C)** Europium (Eu)-enhanced time-resolved fluorescence imaging (TRF) of excised organs from atherosclerotic mice injected with 0.1 mg/kg (based on Eu concentration) of CCTV or VTCC containing 10% (by weight) Eu cryptate-conjugated lipids. **(D)** Magnetic resonance imaging of atherosclerotic plaque (arrows) with CCTV and VTCC containing gadolinium (Gd) lipid-conjugated chelates (DOTA) at 20% (by weight) and injected at 0.1 mg/kg (based on Gd concentration). **(E)** Contrast-to-noise ratio (CNR) quantification of gadolinium-enhanced plaque (Copyright permission from the Ivyspring International Publisher) (Mog et al., 2019).

**FIGURE 13:**
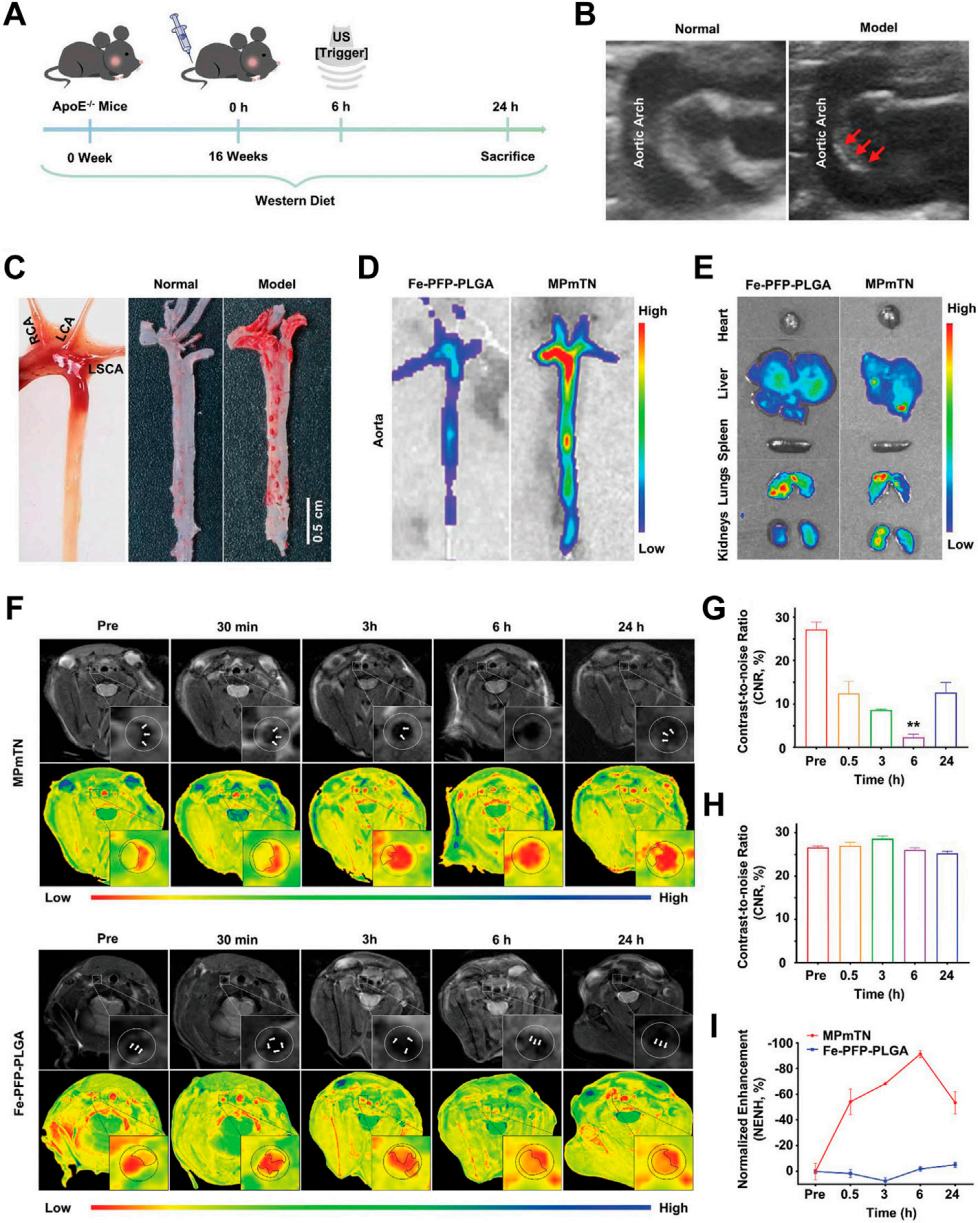
Targeting and MR imaging ability of IPMT-NP *in vivo*. **(A)** Schematic diagram showing the time scale of the *in vivo* animal experiment. **(B)** US images monitored atherosclerotic plaques in the aortic arch of model apoE^−/−^ mice (right, red arrowhead) by comparing with age-matched normal mice (left). **(C)** Gross aortic vessel isolated from age-matched mice (left), representative photographs of Oil Red O-stained aorta form the control group (middle) and the ApoE^−/−^ mice after a 16-week western diet (right). **(D)** Fluorescence images of aorta and **(E)** the extracted organs from mice at 6 h post the injection of Dio-labeled Fe-PFP-PLGA NPs or MPmTNs. **(F)** *T*_*2*_-weighted and pseudo-color images, **(G,H)** CNR, and **(I)** % NENH of aortic plaques at different points after the injection of MPmTNs or the Fe-PFP-PLGA NPs (**p < 0.01) (Copyright permission from the Royal Society of Chemistry) (Gao et al., 2021).

The construction of well-designed, multifunctional nanoparticles not only facilitates imaging but may also play a significant role in assessing the effects of therapies (Lobatto et al., 2010). Recently, Flores et al. developed a precision-engineered nanoparticle system (SWNT-SHP1i) that could disrupt CD47-SIRPα signaling in monocytes and macrophages to promote apoptotic cell clearance within arteriosclerotic lesions, thereby preventing atherosclerosis (Flores et al., 2020). The fluorescent probe Cy5.5 was loaded into the backbone of polyethylene glycol (PEG)-functionalized single-walled carbon nanotubes (SWNTs) to confirm the specific selectivity of SWNTs for circulating Ly-6C^hi^ monocytes and macrophages *in vitro* and *in vivo*. Cy5.5 enabled large-scale single-cell RNA sequencing (scRNA-seq) of leukocytes from SWNT-Cy5.5 to SWNT-SHP1i-treated mice’s aortas by fluorescence-activated cell sorting to assess impacts of chronic efferocytosis stimulation on lesioned macrophages. This experiment greatly expanded the guiding role of fluorescence in efficient assessment of molecule-based therapeutics. Specific targeting of inflammation had the potential to stabilize vulnerable plaques; thus, to assess the therapy effect *via* non-invasive imaging techniques, Maiseyeu constructed self-assembled, peptide-conjugated nanoparticles (CCTV) which possessed antagonist properties toward CC chemokine receptor 2 (CCR2) (Mog et al., 2019). By inhibition of chemotactic migration of primary monocytes and prevention from CCL2-induced actin polymerization, CCTV ameliorated NF-κB activation and downregulated the secondary inflammatory response in macrophages. More importantly, as conjugated with gadolinium or europium cryptates, CCTV enabled targeted MRI or time-resolved fluorescence imaging of atherosclerosis to reveal the chronic inflammatory condition, which provided the real-time imaging information to guide the treatment process (Figure 12). In another study, Gao et al. (2021) proposed a novel strategy called “multifunctional pathology-mapping theranostic nanoplatform (MPmTN)” for the tailored treatment of vulnerable plaques based on the pathological classification (Figure 13). Specifically, MPmTN nanoparticles could target rupture-prone or erosion-prone plaques through specific accumulation mediated by surface-modified PP1 or cyclic RGD (cRGD) peptides. Under therapeutic ultrasound (TUS) exposure, perfluoropentane (PFP) inside the MPmTN would undergo a phase change from nanodroplets to gas microbubbles and induce good imaging properties for US. Incorporation of MR contrast imaging materials (Fe_3_O_4_) inside MPmTN NPs enabled accurate imaging of the two different vulnerable plaques. In addition to the inducible acoustic effects under TUS, MPmTN NPs could also achieve apoptosis of macrophages and disaggregation of activated platelets on vulnerable plaques, which could further promote the therapeutic effects of atherosclerosis. This study provided a potential strategy for personalized treatment of vulnerable plaques based on their pathological nature and a multimodal imaging tool for both the risk stratification and assessment of therapeutic efficacy.

## Conclusion and clinical considerations

Over the past two decades, tremendous progress in imaging technologies has robustly promoted the research studies from imaging the existing atherosclerotic plaque in symptomatic patients to characterizing the asymptomatically vulnerable plaque (Tawakol et al., 2013). However, atherosclerosis remains a major public health problem with devastating consequences. Nowadays, numerous emerged targeted nanoparticles have been involved in the development of more imaging strategies to characterize plaque. Through conjugation with target moieties on the surface, nanoparticles are able to specifically accumulate at atherosclerotic lesions, providing direct read-out of the expression or activity of the vulnerable markers at the molecular level. Such nanoparticle-enhanced molecular imaging strategies can possibly aid early identification of vulnerable plaques most likely to cause cute cardiovascular events and can also help in deciding which therapies are the best candidates for proceeding to clinical trials.

Although clinically approved nanoparticles are still scarce, their excellent performances on plaque imaging in preclinical experiments have opened new avenues for nanomedical atherosclerosis management. However, rather than directly diagnosing the vulnerability index of the plaques, presently reported studies still focus on establishing the relationship between the imaging patterns and the histological markers of plaque progression through quantitatively assessing the accumulated nanoparticles in the plaques. In addition to that, the early attempts with the SPIO nanoparticles in activity of inflammation imaging after myocardial infarction in humans have been suspended, suggesting the clinical translation of nanoparticle-based imaging strategies remains hugely challenging in this field. Also, there still lacks of a deep understanding of the interaction between the nanoparticles and different components in atherosclerotic plaques, and many biological behaviors of nanoparticles need to be further explored: 1) physicochemical interactions at the nano–bio interface should be deeply investigated to optimize the *in vivo* biodistribution, pharmacokinetics, cellular uptake, and intracellular trafficking of nanoparticles; 2) the effects of nanoparticles’ physicochemical properties, such as the particle size, surface potential, and morphology, should be fully researched and optimized to enhance their capability of escaping MPS capture, extravasation and penetration into plaques, targeting efficiency, and subcellular distribution; 3) the biosafety, toxicity, immunogenicity, and clearance of nanoparticles should be systematically evaluated to accelerate their clinical translation.

Notably, the nanotechnology application in cardiovascular disease is certainly not restricted to the injectable nanoparticulate described in this review. Nanotechnology also plays a critical role in tissue engineering, stent design, and early-detection device in aortic aneurysms, thrombolytic therapy, ablation therapy for atrial fibrillation, and other cardiovascular disorders.

## References

[B1] Aboyans V. Ricco J. B. Bartelink M. E. L. Björck M. Brodmann M. Cohnert T. (2018). 2017 ESC guidelines on the diagnosis and treatment of peripheral arterial diseases, in collaboration with the European society for vascular surgery (ESVS): Document covering atherosclerotic disease of extracranial carotid and vertebral, mesenteric, renal, upper and lower extremity arteriesEndorsed by: The European stroke organization (ESO) the task force for the diagnosis and treatment of peripheral arterial diseases of the European society of Cardiology (ESC) and of the European society for vascular surgery (ESVS). Eur. Heart J. 39 (9), 763–816. 10.1093/eurheartj/ehx095 PubMed Abstract | 10.1093/eurheartj/ehx095 | Google Scholar 28886620

[B2] Aizaz M. Moonen R. P. M. van der Pol J. A. J. Prieto C. Botnar R. M. Kooi M. E. (2020). PET/MRI of atherosclerosis. Cardiovasc. Diagn. Ther. 10 (4), 1120–1139. 10.21037/cdt.2020.02.09 PubMed Abstract | 10.21037/cdt.2020.02.09 | Google Scholar 32968664 PMC7487378

[B3] Akyuz A. (2020). Exercise and coronary heart disease. Adv. Exp. Med. Biol. 1228, 169–179. 10.1007/978-981-15-1792-1_11 PubMed Abstract | 10.1007/978-981-15-1792-1_11 | Google Scholar 32342457

[B4] Allahverdian S. Chehroudi A. C. Mcmanus B. M. Abraham T. Francis G. A. (2014). Contribution of intimal smooth muscle cells to cholesterol accumulation and macrophage−like cells in human atherosclerosis. Circulation 129, 1551–1559. 10.1161/CIRCULATIONAHA.113.005015 PubMed Abstract | 10.1161/CIRCULATIONAHA.113.005015 | Google Scholar 24481950

[B5] Alphandéry E. (2022). Nanomaterials as ultrasound theragnostic tools for heart disease treatment/diagnosis. Int. J. Mol. Sci. 23 (3), 1683. 10.3390/ijms23031683 PubMed Abstract | 10.3390/ijms23031683 | Google Scholar 35163604 PMC8835969

[B6] Amirbekian V. Lipinski M. Briley−Saebo K. Amirbekian S. Aguinaldo J. Weinreb D. (2007). Detecting and assessing macrophages *in vivo* to evaluate atherosclerosis noninvasively using molecular MRI. Proc. Natl. Acad. Sci. U. S. A. 104, 961–966. 10.1073/pnas.0606281104 PubMed Abstract | 10.1073/pnas.0606281104 | Google Scholar 17215360 PMC1766334

[B7] Andelovic K. Winter P. Jakob P. M. Bauer W. R. Zernecke A. (2021). Evaluation of plaque characteristics and inflammation using magnetic resonance imaging. Biomedicines 9 (2), 185. 10.3390/biomedicines9020185 PubMed Abstract | 10.3390/biomedicines9020185 | Google Scholar 33673124 PMC7917750

[B8] Aruva M. R. Daviau J. Sharma S. S. Thakur M. L. (2006). Imaging thromboembolism with fibrin−avid 99mTc−peptide: Evaluation in swine. J. Nucl. Med. 47 (1), 155–162. PubMed Abstract | Google Scholar 16391200 PMC1525044

[B9] Barua S. Mitragotri S. (2014). Challenges associated with penetration of nanoparticles across cell and tissue barriers: A review of current status and future prospects. Nano today 9, 223–243. 10.1016/j.nantod.2014.04.008 PubMed Abstract | 10.1016/j.nantod.2014.04.008 | Google Scholar 25132862 PMC4129396

[B10] Beldman T. J. Malinova T. S. Desclos E. Grootemaat A. E. Kluza E. van der Velden S. (2019). Nanoparticle−aided characterization of arterial endothelial architecture during atherosclerosis progression and metabolic therapy. ACS Nano 13 (12), 13759–13774. 10.1021/acsnano.8b08875 PubMed Abstract | 10.1021/acsnano.8b08875 | Google Scholar 31268670 PMC6933811

[B11] Beldman T. J. Senders M. L. Alaarg A. Perez−Medina C. Tang J. Zhao Y. (2017). Hyaluronan nanoparticles selectively target plaque−associated macrophages and improve plaque stability in atherosclerosis. ACS Nano 11 (6), 5785–5799. 10.1021/acsnano.7b01385 PubMed Abstract | 10.1021/acsnano.7b01385 | Google Scholar 28463501 PMC5492212

[B12] Bentzon J. F. Otsuka F. Virmani R. Falk E. (2014). Mechanisms of plaque formation and rupture. Circ. Res. 114 (12), 1852–1866. 10.1161/CIRCRESAHA.114.302721 PubMed Abstract | 10.1161/CIRCRESAHA.114.302721 | Google Scholar 24902970

[B13] Bertrand M. J. Abran M. Maafi F. Busseuil D. Merlet N. Mihalache−Avram T. (2019). *In vivo* near-infrared fluorescence imaging of atherosclerosis using local delivery of novel targeted molecular probes. Sci. Rep. 9 (1), 2670. 10.1038/s41598-019-38970-4 PubMed Abstract | 10.1038/s41598-019-38970-4 | Google Scholar 30804367 PMC6389905

[B14] Binderup T. Duivenvoorden R. Fay F. van Leent M. M. T. Malkus J. Baxter S. (2019). Imaging−assisted nanoimmunotherapy for atherosclerosis in multiple species. Sci. Transl. Med. 11 (506), eaaw7736. 10.1126/scitranslmed.aaw7736 PubMed Abstract | 10.1126/scitranslmed.aaw7736 | Google Scholar 31434756 PMC7328283

[B15] Boring L. Gosling J. Cleary M. Charo I. (1998). Decreased lesion formation in CCR2−/− mice reveals a role for chemokines in the initiation of atherosclerosis. Nature 394 (6696), 894–897. 10.1038/29788 PubMed Abstract | 10.1038/29788 | Google Scholar 9732872

[B16] Bozkurt M. F. Virgolini I. Balogova S. Beheshti M. Rubello D. Decristoforo C. (2017). Guideline for PET/CT imaging of neuroendocrine neoplasms with ^68^Ga−DOTA−conjugated somatostatin receptor targeting peptides and 18F−DOPA. Eur. J. Nucl. Med. Mol. Imaging 44 (9), 1588–1601. 10.1007/s00259-017-3728-y PubMed Abstract | 10.1007/s00259-017-3728-y | Google Scholar 28547177

[B17] Bujold K. Mellal K. Zo Cc Al K. F. Rhainds D. Brissette L. Febbraio M. (2013). EP 80317, a CD36 selective ligand, promotes reverse cholesterol transport in apolipoprotein E−deficient mice. Atherosclerosis 229 (2), 408–414. 10.1016/j.atherosclerosis.2013.05.031 PubMed Abstract | 10.1016/j.atherosclerosis.2013.05.031 | Google Scholar 23880196

[B18] Camaré C. Pucelle M. Nègre−Salvayre A. Salvayre R. (2017). Angiogenesis in the atherosclerotic plaque. Redox Biol. 12, 18–34. 10.1016/j.redox.2017.01.007 PubMed Abstract | 10.1016/j.redox.2017.01.007 | Google Scholar 28212521 PMC5312547

[B19] Chadderdon S. M. Belcik J. T. Bader L. Kirigiti M. A. Peters D. M. Kievit P. (2014). Proinflammatory endothelial activation detected by molecular imaging in obese nonhuman primates coincides with onset of insulin resistance and progressively increases with duration of insulin resistance. Circulation 129 (4), 471–478. 10.1161/CIRCULATIONAHA.113.003645 PubMed Abstract | 10.1161/CIRCULATIONAHA.113.003645 | Google Scholar 24163066 PMC3909516

[B20] Chen F. Wang G. Griffin J. I. Brenneman B. Banda N. K. Holers V. M. (2017). Complement proteins bind to nanoparticle protein corona and undergo dynamic exchange *in vivo* . Nat. Nanotechnol. 12, 387–393. 10.1038/nnano.2016.269 PubMed Abstract | 10.1038/nnano.2016.269 | Google Scholar 27992410 PMC5617637

[B21] Chen J. Zhang X. Millican R. Sherwood J. Jun H. W. Jo H. (2021). Recent advances in nanomaterials for therapy and diagnosis for atherosclerosis. Adv. Drug Deliv. Rev. 170, 142–199. 10.1016/j.addr.2021.01.005 PubMed Abstract | 10.1016/j.addr.2021.01.005 | Google Scholar 33428994 PMC7981266

[B22] Cheng D. Li X. Zhang C. Tan H. Wang C. Pang L. (2015). Detection of vulnerable atherosclerosis plaques with a dual−modal single−photon−emission computed tomography/magnetic resonance imaging probe targeting apoptotic macrophages. ACS Appl. Mat. Interfaces 7, 2847–2855. 10.1021/am508118x PubMed Abstract | 10.1021/am508118x | Google Scholar 25569777

[B23] Chiu J. J. Chien S. (2011). Effects of disturbed flow on vascular endothelium: Pathophysiological basis and clinical perspectives. Physiol. Rev. 91 (1), 327–387. 10.1152/physrev.00047.2009 PubMed Abstract | 10.1152/physrev.00047.2009 | Google Scholar 21248169 PMC3844671

[B24] Chung E. J. Mlinar L. B. Nord K. Sugimoto M. J. Wonder E. Alenghat F. J. (2014). Monocyte−targeting supramolecular micellar assemblies: A molecular diagnostic tool for atherosclerosis. Adv. Healthc. Mat. 4 (3), 367–376. 10.1002/adhm.201400336 PubMed Abstract | 10.1002/adhm.201400336 | Google Scholar PMC4336846 25156590

[B25] Combadiere C. Potteaux S. Rodero M. Simon T. Pezard A. Esposito B. (2008). Combined inhibition of CCL2, CX3CR1, and CCR5 abrogates ly6chi and ly6clo monocytosis and almost abolishes atherosclerosis in hypercholesterolemic mice. Circulation 117, 1649–1657. 10.1161/CIRCULATIONAHA.107.745091 PubMed Abstract | 10.1161/CIRCULATIONAHA.107.745091 | Google Scholar 18347211

[B26] Cormode D. P. Chandrasekar R. Delshad A. Briley−Saebo K. C. Calcagno C. Barazza A. (2009). Comparison of synthetic high density lipoprotein (HDL) contrast agents for mr imaging of atherosclerosis. Bioconjug. Chem. 20 (5), 937–943. 10.1021/bc800520d PubMed Abstract | 10.1021/bc800520d | Google Scholar 19378935 PMC2765543

[B27] Cormode D. P. Roessl E. Thran A. Skajaa T. Gordon R. E. Schlomka J. P. (2010). Atherosclerotic plaque composition: Analysis with multicolor CT and targeted gold nanoparticles. Radiology 256 (3), 774–782. 10.1148/radiol.10092473 PubMed Abstract | 10.1148/radiol.10092473 | Google Scholar 20668118 PMC2923725

[B28] Cormode D. P. Skajaa T. van Schooneveld M. M. Koole R. Jarzyna P. Lobatto M. E. (2008). Nanocrystal core high−density lipoproteins: A multimodality contrast agent platform. Nano Lett. 8 (11), 3715–3723. 10.1021/nl801958b PubMed Abstract | 10.1021/nl801958b | Google Scholar 18939808 PMC2629801

[B29] Danila D. Partha R. Elrod D. B. Lackey M. Casscells S. W. Conyers J. L. (2009). Antibody−labeled liposomes for CT imaging of atherosclerotic plaques: *In vitro* investigation of an anti−ICAM antibody−labeled liposome containing iohexol for molecular imaging of atherosclerotic plaques via computed tomography. Tex Heart Inst. J. 36, 393–403. PubMed Abstract | Google Scholar 19876414 PMC2763481

[B30] de Weert T. T. Ouhlous M. Meijering E. Zondervan P. E. Hendriks J. M. van Sambeek M. R. (2006). *In vivo* characterization and quantification of atherosclerotic carotid plaque components with multidetector computed tomography and histopathological correlation. Arterioscler. Thromb. Vasc. Biol. 26, 2366–2372. 10.1161/01.ATV.0000240518.90124.57 PubMed Abstract | 10.1161/01.ATV.0000240518.90124.57 | Google Scholar 16902158

[B31] Deguchi J. O. Aikawa M. Tung C. H. Aikawa E. Kim D. E. Ntziachristos V. (2006). Inflammation in atherosclerosis: Visualizing matrix metalloproteinase action in macrophages *in vivo* . Circulation 114 (1), 55–62. 10.1161/CIRCULATIONAHA.106.619056 PubMed Abstract | 10.1161/CIRCULATIONAHA.106.619056 | Google Scholar 16801460

[B32] Ding J. Wang Y. Ma M. Zhang Y. Lu S. Jiang Y. (2013). CT/fluorescence dual−modal nanoemulsion platform for investigating atherosclerotic plaques. Biomaterials 34, 209–216. 10.1016/j.biomaterials.2012.09.025 PubMed Abstract | 10.1016/j.biomaterials.2012.09.025 | Google Scholar 23069709

[B33] Douketis J. D. Ginsberg J. S. Haley S. Julian J. Dwyer M. Levine M. (2012). Accuracy and safety of ^99^mTc−labeled anti−D−dimer (DI−80B3) fab' fragments (ThromboView) in the diagnosis of deep vein thrombosis: A phase II study. Thromb. Res. 130, 381–389. 10.1016/j.thromres.2012.05.011 PubMed Abstract | 10.1016/j.thromres.2012.05.011 | Google Scholar 22658414

[B34] Du B. Jiang X. Das A. Zhou Q. Yu M. Jin R. (2017). Glomerular barrier behaves as an atomically precise bandpass filter in a sub−nanometre regime. Nat. Nanotechnol. 12 (11), 1096–1102. 10.1038/nnano.2017.170 PubMed Abstract | 10.1038/nnano.2017.170 | Google Scholar 28892099 PMC5679252

[B35] Duivenvoorden R. Tang J. Cormode D. P. Mieszawska A. J. Izquierdo−Garcia D. Ozcan C. (2014). A statin−loaded reconstituted high−density lipoprotein nanoparticle inhibits atherosclerotic plaque inflammation. Nat. Commun. 5, 3065. 10.1038/ncomms4065 PubMed Abstract | 10.1038/ncomms4065 | Google Scholar 24445279 PMC4001802

[B36] Fei Y. Wei Y. Xiang L. Hongmei L. Xiang N. Lisi X. (2015). Magnetic resonance imaging of atherosclerosis using CD81−targeted microparticles of iron oxide in mice. Biomed. Res. Int. 2015, 758616. 10.1155/2015/758616 PubMed Abstract | 10.1155/2015/758616 | Google Scholar 26266263 PMC4523685

[B37] Fei Y. Yu S. Yang M. Meiying W. Zhiting D. Shuai L. (2018). Ultrasound molecular imaging of atherosclerosis for early diagnosis and therapeutic evaluation through leucocyte−like multiple targeted microbubbles. Theranostics 8, 1879–1891. 10.7150/thno.22070 PubMed Abstract | 10.7150/thno.22070 | Google Scholar 29556362 PMC5858506

[B38] Fleischmann D. Goepferich A. (2021). General sites of nanoparticle biodistribution as a novel opportunity for nanomedicine. Eur. J. Pharm. Biopharm. 166, 44–60. 10.1016/j.ejpb.2021.05.027 PubMed Abstract | 10.1016/j.ejpb.2021.05.027 | Google Scholar 34087354

[B39] Flores A. M. Hosseini−Nassab N. Jarr K. U. Ye J. Zhu X. Wirka R. (2020). Pro−efferocytic nanoparticles are specifically taken up by lesional macrophages and prevent atherosclerosis. Nat. Nanotechnol. 15, 154–161. 10.1038/s41565-019-0619-3 PubMed Abstract | 10.1038/s41565-019-0619-3 | Google Scholar 31988506 PMC7254969

[B40] Gao B. Xu J. Zhou J. Zhang H. Yang R. Wang H. (2021). Multifunctional pathology−mapping theranostic nanoplatforms for US/MR imaging and ultrasound therapy of atherosclerosis. Nanoscale 13 (18), 8623–8638. 10.1039/d1nr01096d PubMed Abstract | 10.1039/d1nr01096d | Google Scholar 33929480

[B41] Garnacho C. Serrano D. Muro S. (2012). A fibrinogen−derived peptide provides intercellular adhesion molecule−1−specific targeting and intraendothelial transport of polymer nanocarriers in human cell cultures and mice. J. Pharmacol. Exp. Ther. 340 (3), 638–647. 10.1124/jpet.111.185579 PubMed Abstract | 10.1124/jpet.111.185579 | Google Scholar 22160267 PMC3286316

[B42] Ge X. Cui H. Kong J. Lu S. Y. Zhan R. Gao J. (2020). A non-invasive nanoprobe for *in vivo* photoacoustic imaging of vulnerable atherosclerotic plaque. Adv. Mat. 32 (38), e2000037. 10.1002/adma.202000037 10.1002/adma.202000037 | Google Scholar 32803803

[B43] Gifani M. Eddins D. J. Kosuge H. Zhang Y. Paluri S. L. A. Larson T. (2021). Ultra−selective carbon nanotubes for photoacoustic imaging of inflamed atherosclerotic plaques. Adv. Funct. Mat. 31 (37), 2101005. 10.1002/adfm.202101005 10.1002/adfm.202101005 | Google Scholar PMC8559995 34733130

[B44] Gong Y. Cao C. Guo Y. Chang B. Sheng Z. Shen W. (2021). Quantification of intracranial arterial stenotic degree evaluated by high−resolution vessel wall imaging and time−of−flight MR angiography: Reproducibility, and diagnostic agreement with DSA. Eur. Radiol. 31 (8), 5479–5489. 10.1007/s00330-021-07719-x PubMed Abstract | 10.1007/s00330-021-07719-x | Google Scholar 33585995

[B45] Group W. Naylor A. R. Ricco J. B. Borst G. D. Debus S. Haro J. D. (2017). Management of atherosclerotic carotid and vertebral artery disease: 2017 clinical practice guidelines of the European society for vascular surgery (ESVS). Eur. J. Vasc. Endovasc. 55 (1), 142–143. 10.1016/j.ejvs.2017.06.021 10.1016/j.ejvs.2017.06.021 | Google Scholar 29221934

[B46] Guo B. Li Z. Tu P. Tang H. Tu Y. (2021). Molecular imaging and non−molecular imaging of atherosclerotic plaque thrombosis. Front. Cardiovasc. Med. 8, 692915. 10.3389/fcvm.2021.692915 PubMed Abstract | 10.3389/fcvm.2021.692915 | Google Scholar 34291095 PMC8286992

[B47] Harris T. D. Rajopadhye M. Damphousse P. R. Glowacka D. Yu K. Bourque J. P. (1996). Tc−99m−labeled fibrinogen receptor antagonists: Design and synthesis of cyclic RGD peptides for the detection of thrombi. Bioorg. Med. Chem. Lett. 6 (15), 1741–1746. 10.1016/0960-894X(96)00282-X 10.1016/0960-894X(96)00282-X | Google Scholar

[B48] Hartog A. Bovens S. M. Koning W. Hendrikse J. Borst G. Moll F. L. (2012). Current status of clinical magnetic resonance imaging for plaque characterisation in patients with carotid artery stenosis. Eur. J. Vasc. Endovasc. Surg. 45 (1), 7–21. 10.1016/j.ejvs.2012.10.022 PubMed Abstract | 10.1016/j.ejvs.2012.10.022 | Google Scholar 23200607

[B49] Hatsukami T. S. Ross R. Polissar N. L. Yuan C. (2000). Visualization of fibrous cap thickness and rupture in human atherosclerotic carotid plaque *in vivo* with high−resolution magnetic resonance imaging. Circulation 102 (9), 959–964. 10.1161/01.cir.102.9.959 PubMed Abstract | 10.1161/01.cir.102.9.959 | Google Scholar 10961958

[B50] Hecht H. S. (2015). Coronary artery calcium scanning: Past, present, and future. JACC. Cardiovasc. Imaging 8 (5), 579–596. 10.1016/j.jcmg.2015.02.006 PubMed Abstract | 10.1016/j.jcmg.2015.02.006 | Google Scholar 25937196

[B51] Hu B. Boakye−Yiadom K. O. Yu W. Yuan Z. W. Ho W. Xu X. (2020). Nanomedicine approaches for advanced diagnosis and treatment of atherosclerosis and related ischemic diseases. Adv. Healthc. Mat. 9 (16), e2000336. 10.1002/adhm.202000336 10.1002/adhm.202000336 | Google Scholar 32597562

[B52] Hu J. Ortgies D. H. Martín Rodríguez E. Rivero F. Aguilar Torres R. Alfonso F. (2018). Optical nanoparticles for cardiovascular imaging. Adv. Opt. Mater. 6 (22), 1800626. 10.1002/adom.201800626 10.1002/adom.201800626 | Google Scholar

[B53] Hyafil F. Cornily J. C. Feig J. E. Gordon R. Vucic E. Amirbekian V. (2007). Noninvasive detection of macrophages using a nanoparticulate contrast agent for computed tomography. Nat. Med. 13 (5), 636–641. 10.1038/nm1571 PubMed Abstract | 10.1038/nm1571 | Google Scholar 17417649

[B54] Hyafil F. Schindler A. Sepp D. Obenhuber T. Bayer−Karpinska A. Boeckh−Behrens T. (2016). High-risk plaque features can be detected in non-stenotic carotid plaques of patients with ischaemic stroke classified as cryptogenic using combined (18)F-FDG PET/MR imaging. Eur. J. Nucl. Med. Mol. Imaging 43 (2), 270–279. 10.1007/s00259-015-3201-8 PubMed Abstract | 10.1007/s00259-015-3201-8 | Google Scholar 26433367

[B55] Jaffer F. A. Tung C. H. Gerszten R. E. Weissleder R. (2002). *In vivo* imaging of thrombin activity in experimental thrombi with thrombin−sensitive near−infrared molecular probe. Arterioscler. Thromb. Vasc. Biol. 22 (11), 1929–1935. 10.1161/01.atv.0000033089.56970.2d PubMed Abstract | 10.1161/01.atv.0000033089.56970.2d | Google Scholar 12426227

[B56] Jaffer F. A. Tung C. H. Wykrzykowska J. J. Ho N. H. Houng A. K. Reed G. L. (2004). Molecular imaging of factor XIIIA activity in thrombosis using a novel, near−infrared fluorescent contrast agent that covalently links to thrombi. Circulation 110 (2), 170–176. 10.1161/01.CIR.0000134484.11052.44 PubMed Abstract | 10.1161/01.CIR.0000134484.11052.44 | Google Scholar 15210587

[B57] Kataoka Y. Wolski K. Uno K. Puri R. Tuzcu E. M. Nissen S. E. (2012). Spotty calcification as a marker of accelerated progression of coronary atherosclerosis: Insights from serial intravascular ultrasound. J. Am. Coll. Cardiol. 59 (18), 1592–1597. 10.1016/j.jacc.2012.03.012 PubMed Abstract | 10.1016/j.jacc.2012.03.012 | Google Scholar 22538329

[B58] Keliher E. J. Ye Y. X. Wojtkiewicz G. R. Aguirre A. D. Tricot B. Senders M. L. (2017). Polyglucose nanoparticles with renal elimination and macrophage avidity facilitate PET imaging in ischaemic heart disease. Nat. Commun. 8, 14064. 10.1038/ncomms14064 PubMed Abstract | 10.1038/ncomms14064 | Google Scholar 28091604 PMC5241815

[B59] Kelly K. A. Allport J. R. Tsourkas A. Shinde−Patil V. R. Josephson L. Weissleder R. (2005). Detection of vascular adhesion molecule−1 expression using a novel multimodal nanoparticle. Circ. Res. 96 (3), 327–336. 10.1161/01.RES.0000155722.17881.dd PubMed Abstract | 10.1161/01.RES.0000155722.17881.dd | Google Scholar 15653572

[B60] Kharlamov A. N. Tyurnina A. E. Veselova V. S. Kovtun O. P. Shur V. Y. Gabinsky J. L. (2015). Silica–gold nanoparticles for atheroprotective management of plaques: Results of the NANOM−FIM trial. Nanoscale 7, 8003–8015. 10.1039/c5nr01050k PubMed Abstract | 10.1039/c5nr01050k | Google Scholar 25864858

[B61] Kim D. Park S. Lee J. H. Jeong Y. Y. Jon S. (2007). Antibiofouling polymer−coated gold nanoparticles as a contrast agent for *in vivo* x−ray computed tomography imaging. J. Am. Chem. Soc. 129 (41), 7661–7665. 10.1021/ja071471p PubMed Abstract | 10.1021/ja071471p | Google Scholar 17530850

[B62] Kim J. B. Park K. Ryu J. Lee J. J. Lee M. W. Cho H. S. (2016). Intravascular optical imaging of high−risk plaques *in vivo* by targeting macrophage mannose receptors. Sci. Rep. 6, 22608. 10.1038/srep22608 PubMed Abstract | 10.1038/srep22608 | Google Scholar 26948523 PMC4780083

[B63] Kim S. E. Roberts J. A. Eisenmenger L. B. Aldred B. W. Jamil O. Bolster B. D. (2017). Motion−insensitive carotid intraplaque hemorrhage imaging using 3D inversion recovery preparation stack of stars (IR−prep SOS) technique. J. Magn. Reson. Imaging 45, 410–417. 10.1002/jmri.25365 PubMed Abstract | 10.1002/jmri.25365 | Google Scholar 27383756 PMC5441883

[B64] Kim Y. Lobatto M. E. Kawahara T. Chung B. L. Mieszawska A. J. Sanchez−Gaytan B. L. (2014). Probing nanoparticle translocation across the permeable endothelium in experimental atherosclerosis. Proc. Natl. Acad. Sci. U. S. A. 111 (3), 1078–1083. 10.1073/pnas.1322725111 PubMed Abstract | 10.1073/pnas.1322725111 | Google Scholar 24395808 PMC3903216

[B65] Klink A. Lancelot E. Ballet S. Vucic E. Fabre J. E. Gonzalez W. (2010). Magnetic resonance molecular imaging of thrombosis in an arachidonic acid mouse model using an activated platelet targeted probe. Arterioscler. Thromb. Vasc. Biol. 30 (3), 403–410. 10.1161/ATVBAHA.109.198556 PubMed Abstract | 10.1161/ATVBAHA.109.198556 | Google Scholar 20139362 PMC2864133

[B66] Kolodgie F. D. Narula J. Yuan C. Burke A. P. Finn A. V. Virmani R. (2007). Elimination of neoangiogenesis for plaque stabilization: Is there a role for local drug therapy? J. Am. Coll. Cardiol. 49 (21), 2093–2101. 10.1016/j.jacc.2006.10.083 PubMed Abstract | 10.1016/j.jacc.2006.10.083 | Google Scholar 17531658

[B67] Kooi M. E. Cappendijk V. C. Cleutjens K. B. J. M. Kessels A. G. H. Kitslaar P. J. E. H. M. Borgers M. (2003)., 107. CC, 2453–2458. 10.1161/01.CIR.0000068315.98705 Accumulation of Ultrasmall superparamagnetic particles of iron oxide in human atherosclerotic plaques can be detected by *in vivo* magnetic resonance imaging Circulation 19 PubMed Abstract | 10.1161/01.CIR.0000068315.98705 | Google Scholar 12719280

[B68] Kwiecinski J. Lassen M. L. Slomka P. J. (2021). Advances in quantitative analysis of (18)F−Sodium fluoride coronary imaging. Mol. Imaging 2021, 8849429. 10.1155/2021/8849429 PubMed Abstract | 10.1155/2021/8849429 | Google Scholar 33746631 PMC7953548

[B69] Kwon S. P. Jeon S. Lee S. H. Yoon H. Y. Ryu J. H. Choi D. (2018). Thrombin−activatable fluorescent peptide incorporated gold nanoparticles for dual optical/computed tomography thrombus imaging. Biomaterials 150, 125–136. 10.1016/j.biomaterials.2017.10.017 PubMed Abstract | 10.1016/j.biomaterials.2017.10.017 | Google Scholar 29035738

[B70] Lameijer M. Binderup T. Leent M. V. Senders M. L. Fay F. Malkus J. (2018). Efficacy and safety assessment of a TRAF6−targeted nanoimmunotherapy in atherosclerotic mice and non−human primates. Nat. Biomed. Eng. 2 (5), 279–292. 10.1038/s41551-018-0221-2 PubMed Abstract | 10.1038/s41551-018-0221-2 | Google Scholar 30936448 PMC6447057

[B71] Lecaille F. Weidauer E. Juliano M. A. Brömme D. Lalmanach G. (2003). Probing cathepsin K activity with a selective substrate spanning its active site. Biochem. J. 375 (2), 307–312. 10.1042/BJ20030468 PubMed Abstract | 10.1042/BJ20030468 | Google Scholar 12837132 PMC1223680

[B72] Lee G. Y. Kim J. H. Oh G. T. Lee B. H. Kwon I. C. Kim I. S. (2011). Molecular targeting of atherosclerotic plaques by a stabilin−2−specific peptide ligand. J. Control. Release 155 (2), 211–217. 10.1016/j.jconrel.2011.07.010 PubMed Abstract | 10.1016/j.jconrel.2011.07.010 | Google Scholar 21781994

[B73] Li P. Jin L. Feng L. Wang Y. Yang R. (2021). ICAM−1−carrying targeted nano contrast agent for evaluating inflammatory injury in rabbits with atherosclerosis. Sci. Rep. 11 (1), 16508. 10.1038/s41598-021-96042-y PubMed Abstract | 10.1038/s41598-021-96042-y | Google Scholar 34389762 PMC8363608

[B74] Li X. Wang C. Tan H. Cheng L. Liu G. Yang Y. (2016). Gold nanoparticles−based SPECT/CT imaging probe targeting for vulnerable atherosclerosis plaques. Biomaterials 108, 71–80. 10.1016/j.biomaterials.2016.08.048 PubMed Abstract | 10.1016/j.biomaterials.2016.08.048 | Google Scholar 27619241

[B75] Liang M. Hui T. Zhou J. Wang T. Duan D. Fan K. (2018). Bioengineered H−ferritin nanocages for quantitative imaging of vulnerable plaques in atherosclerosis. Acs Nano 12, 9300–9308. 10.1021/acsnano.8b04158 PubMed Abstract | 10.1021/acsnano.8b04158 | Google Scholar 30165015

[B76] Libby P. (2021). The changing nature of atherosclerosis: What we thought we knew, what we think we know, and what we have to learn. Eur. Heart J. 42 (47), 4781–4782. 10.1093/eurheartj/ehab438 PubMed Abstract | 10.1093/eurheartj/ehab438 | Google Scholar 34905600 PMC8670782

[B77] Lipinski M. J. Frias J. C. Amirbekian V. Briley−Saebo K. C. Mani V. Samber D. (2009). Macrophage−specific lipid−based nanoparticles improve cardiac magnetic resonance detection and characterization of human atherosclerosis. JACC. Cardiovasc. Imaging 2 (5), 637–647. 10.1016/j.jcmg.2008.08.009 PubMed Abstract | 10.1016/j.jcmg.2008.08.009 | Google Scholar 19442953 PMC2756539

[B78] Lister−James J. Knight L. C. Maurer A. H. Bush L. R. Dean R. T. (1996). Thrombus imaging with a technetium−99m−labeled, activated platelet receptor−binding peptide. J. Nucl. Med. 37 (5), 775–781. PubMed Abstract | Google Scholar 8965144

[B79] Lister−James J. Vallabhajosula S. Moyer B. R. Pearson D. A. Dean R. T. De Rosch M. A. (1997). Pre−clinical evaluation of technetium−99m platelet receptor−binding peptide. J. Nucl. Med. 38 (1), 105–111. PubMed Abstract | Google Scholar 8998163

[B80] Liu J. Zhang P. Liu P. Zhao Y. Gao S. Tan K. (2012). Endothelial adhesion of targeted microbubbles in both small and great vessels using ultrasound radiation force. Mol. Imaging 11 (1), 7290.2011.00027–66. 10.2310/7290.2011.00027 PubMed Abstract | 10.2310/7290.2011.00027 | Google Scholar 22418028

[B81] Lobatto M. E. Fayad Z. A. Silvera S. Vucic E. Calcagno C. Mani V. (2010). Multimodal clinical imaging to longitudinally assess a nanomedical anti−inflammatory treatment in experimental atherosclerosis. Mol. Pharm. 7 (6), 2020–2029. 10.1021/mp100309y PubMed Abstract | 10.1021/mp100309y | Google Scholar 21028895 PMC3345199

[B82] Lobatto M. E. Fuster V. Fayad Z. A. Mulder W. J. (2011). Perspectives and opportunities for nanomedicine in the management of atherosclerosis. Nat. Rev. Drug Discov. 10 (11), 835–852. 10.1038/nrd3578 PubMed Abstract | 10.1038/nrd3578 | Google Scholar 22015921 PMC3623275

[B83] Louie A. (2010). Multimodality imaging probes: Design and challenges. Chem. Rev. 110 (5), 3146–3195. 10.1021/cr9003538 PubMed Abstract | 10.1021/cr9003538 | Google Scholar 20225900 PMC2878382

[B84] Luehmann H. P. Pressly E. Detering L. Wang C. Pierce R. Woodard P. K. (2014). PET/CT imaging of chemokine receptor ccr5 in vascular injury model using targeted nanoparticle. J. Nucl. Med. 55 (4), 629–634. 10.2967/jnumed.113.132001 PubMed Abstract | 10.2967/jnumed.113.132001 | Google Scholar 24591489 PMC4255944

[B85] Ma Q. Fan Q. Han X. Dong Z. Xu J. Bai J. (2020). Platelet−derived extracellular vesicles to target plaque inflammation for effective anti−atherosclerotic therapy. J. Control. Release 329, 445–453. 10.1016/j.jconrel.2020.11.064 PubMed Abstract | 10.1016/j.jconrel.2020.11.064 | Google Scholar 33285103

[B86] Ma Y. Xu L. Yin B. Shang J. Chen F. Xu J. (2021). Ratiometric semiconducting polymer nanoparticle for reliable photoacoustic imaging of pneumonia−induced vulnerable atherosclerotic plaque *in vivo* . Nano Lett. 21 (10), 4484–4493. 10.1021/acs.nanolett.1c01359 PubMed Abstract | 10.1021/acs.nanolett.1c01359 | Google Scholar 33978427

[B87] Macfarlane D. Socrates A. Eisenberg P. Larcos G. Roach P. Gerometta M. (2009). Imaging of deep venous thrombosis in patients using a radiolabelled anti−D−dimer Fab′fragment ( 99m Tc−DI−DD3B6/22−80B3): Results of a phase I trial. Eur. J. Nucl. Med. Mol. Imaging 36 (2), 250–259. 10.1007/s00259-008-0934-7 PubMed Abstract | 10.1007/s00259-008-0934-7 | Google Scholar 18800218

[B88] Magnus Bäck Arif Yurdagul Ira Tabas Oorni K. Kovanen P. T. (2019). Inflammation and its resolution in atherosclerosis: Mediators and therapeutic opportunities. Nat. Rev. Cardiol. 16, 389–406. 10.1038/s41569-019-0169-2 PubMed Abstract | 10.1038/s41569-019-0169-2 | Google Scholar 30846875 PMC6727648

[B89] Maldiney T. Bessière A. Seguin J. Teston E. Sharma S. K. Viana B. (2014). The *in vivo* activation of persistent nanophosphors for optical imaging of vascularization, tumours and grafted cells. Nat. Mat. 13 (4), 418–426. 10.1038/nmat3908 PubMed Abstract | 10.1038/nmat3908 | Google Scholar 24651431

[B90] Michalska M. Machtoub L. Manthey H. D. Bauer E. Herold V. Krohne G. (2012). Visualization of vascular inflammation in the atherosclerotic mouse by ultrasmall superparamagnetic iron oxide vascular cell adhesion molecule−1–specific nanoparticles. Arterioscler. Thromb. Vasc. Biol. 32 (10), 2350–2357. 10.1161/ATVBAHA.112.255224 PubMed Abstract | 10.1161/ATVBAHA.112.255224 | Google Scholar 22879583

[B91] Mog B. Asase C. Chaplin A. Gao H. Maiseyeu A. (2019). Nano−Antagonist alleviates inflammation and allows for MRI of atherosclerosis. Nanotheranostics 3 (4), 342–355. 10.7150/ntno.37391 PubMed Abstract | 10.7150/ntno.37391 | Google Scholar 31723548 PMC6838142

[B92] Moore K. J. Tabas I. (2011). Macrophages in the pathogenesis of atherosclerosis. Cell. 145, 341–355. 10.1016/j.cell.2011.04.005 PubMed Abstract | 10.1016/j.cell.2011.04.005 | Google Scholar 21529710 PMC3111065

[B93] Moreno P. R. Purushothaman K. R. Zias E. Sanz J. Fuster V. Kacher D. F. (2006). Neovascularization in Human Atherosclerosis. Curr. Mol. Med. 6, 457–477. 10.2174/156652406778018635 PubMed Abstract | 10.2174/156652406778018635 | Google Scholar 16918368

[B163] Morishige K. Kacher D. F. Libby P. Josephson L. Ganz P. Weissleder R. (2010). High–Resolution magnetic resonance imaging enhanced with superparamagnetic nanoparticles measures macrophage burden atherosclerosis. Circulation 122 (17), 1707–1715. 10.1161/CIRCULATIONAHA.109.89180 PubMed Abstract | 10.1161/CIRCULATIONAHA.109.89180 | Google Scholar 20937980 PMC3003265

[B94] Mousa S. A. Bozarth J. M. Forsythe M. S. Lorelli W. Thoolen M. J. Ramachandran N. (1993). Antiplatelet efficacy and specificity of DMP728, a novel platelet GPIIb/IIIa receptor antagonist. Cardiology 83 (5−6), 374–382. 10.1159/000175994 PubMed Abstract | 10.1159/000175994 | Google Scholar 7509257

[B95] Naghavi M. Libby P. Falk E. Casscells S. W. Litovsky S. Rumberger J. (2003). From vulnerable plaque to vulnerable patient: A call for new definitions and risk assessment strategies: Part I. Circulation 108 (14), 1664–1672. 10.1161/01.CIR.0000087480.94275.97 PubMed Abstract | 10.1161/01.CIR.0000087480.94275.97 | Google Scholar 14530185

[B96] Nahrendorf M. Jaffer F. A. Kelly K. A. Sosnovik D. E. Aikawa E. Libby P. (2006). Noninvasive vascular cell adhesion molecule−1 imaging identifies inflammatory activation of cells in atherosclerosis. Circulation 114 (14), 1504–1511. 10.1161/CIRCULATIONAHA.106.646380 PubMed Abstract | 10.1161/CIRCULATIONAHA.106.646380 | Google Scholar 17000904

[B97] Narita Y. Shimizu K. Ikemoto K. Uchino R. Kosugi M. Maess M. B. (2019). Macrophage−targeted, enzyme−triggered fluorescence switch−on system for detection of embolism−vulnerable atherosclerotic plaques. J. Control. Release 302, 105–115. 10.1016/j.jconrel.2019.03.025 PubMed Abstract | 10.1016/j.jconrel.2019.03.025 | Google Scholar 30936020

[B98] Nasr S. H. Rashidijahanabad Z. Ramadan S. Kauffman N. Parameswaran N. Zinn K. R. (2020). Effective atherosclerotic plaque inflammation inhibition with targeted drug delivery by hyaluronan conjugated atorvastatin nanoparticles. Nanoscale 12 (17), 9541–9556. 10.1039/d0nr00308e PubMed Abstract | 10.1039/d0nr00308e | Google Scholar 32314997 PMC7234819

[B99] Neubauer P. M. Caruthers A. M. Harris S. D. Robertson T. D. Williams J. D. Schmieder T. A. (2006). Endothelial alpha(v)beta3 integrin−targeted fumagillin nanoparticles inhibit angiogenesis in atherosclerosis. Arterioscler. Thromb. Vasc. Biol. 26 (9), 2103–2109. 10.1161/01.ATV.0000235724.11299.76 PubMed Abstract | 10.1161/01.ATV.0000235724.11299.76 | Google Scholar 16825592

[B100] Ouimet T. Lancelot E. Hyafil F. Rienzo M. Ballet S. Lemaitre M. (2012). Molecular and cellular targets of the MRI contrast agent p947 for atherosclerosis imaging. Mol. Pharm. 9 (4), 850–861. 10.1021/mp2003863 PubMed Abstract | 10.1021/mp2003863 | Google Scholar 22352457

[B101] Overoye−Chan K. Koerner S. Looby R. J. Kolodziej A. F. Zech S. G. Deng Q. (2008). EP−2104R: A fibrin−specific gadolinium−Based MRI contrast agent for detection of thrombus. J. Am. Chem. Soc. 130 (18), 6025–6039. 10.1021/ja800834y PubMed Abstract | 10.1021/ja800834y | Google Scholar 18393503

[B102] Park K. Hong H. Y. Moon H. J. Lee B. H. Kim I. S. Kwon I. C. (2008). A new atherosclerotic lesion probe based on hydrophobically modified chitosan nanoparticles functionalized by the atherosclerotic plaque targeted peptides. J. Control. Release 128 (3), 217–223. 10.1016/j.jconrel.2008.03.019 PubMed Abstract | 10.1016/j.jconrel.2008.03.019 | Google Scholar 18457896

[B103] Paulis L. E. Jacobs I. Akker N. Geelen T. Strijkers G. J. Starmans L. W. E. (2012). Targeting of ICAM−1 on vascular endothelium under static and shear stress conditions using a liposomal Gd−based MRI contrast agent. J. Nanobiotechnology 10 (1), 25. 10.1186/1477-3155-10-25 PubMed Abstract | 10.1186/1477-3155-10-25 | Google Scholar 22716048 PMC3563567

[B104] Pérez−Medina C. Binderup T. Lobatto M. E. Tang J. Calcagno C. Giesen L. (2016). *In vivo* PET imaging of HDL in multiple atherosclerosis models. JACC. Cardiovasc. Imaging 9 (9), 950–961. 10.1016/j.jcmg.2016.01.020 PubMed Abstract | 10.1016/j.jcmg.2016.01.020 | Google Scholar 27236528 PMC5589956

[B105] Qiao R. Qiao H. Zhang Y. Wang Y. Chi C. Tian J. (2017). Molecular imaging of vulnerable atherosclerotic plaques *in vivo* with osteopontin−specific upconversion nanoprobes. Acs Nano 11 (2), 1816–1825. 10.1021/acsnano.6b07842 PubMed Abstract | 10.1021/acsnano.6b07842 | Google Scholar 28121134

[B106] Ramirez−Carracedo R. Tesoro L. Hernandez I. Diez−Mata J. Filice M. Toro R. (2018). Non−Invasive detection of extracellular matrix metalloproteinase inducer EMMPRIN, a new therapeutic target against atherosclerosis, inhibited by endothelial nitric oxide. Int. J. Mol. Sci. 19 (10), 3248. 10.3390/ijms19103248 10.3390/ijms19103248 | Google Scholar PMC6214015 30347750

[B107] Rollett A. Reiter T. Nogueira P. Cardinale M. Loureiro A. Gomes A. (2012). Folic acid−functionalized human serum albumin nanocapsules for targeted drug delivery to chronically activated macrophages. Int. J. Pharm. 427 (2), 460–466. 10.1016/j.ijpharm.2012.02.028 PubMed Abstract | 10.1016/j.ijpharm.2012.02.028 | Google Scholar 22374516

[B108] Rucher G. Cameliere L. Fendri J. Anfray A. Abbas A. Kamel S. (2019). Molecular imaging of endothelial activation and mineralization in a mouse model of accelerated atherosclerosis. EJNMMI Res. 9 (1), 80. 10.1186/s13550-019-0550-5 PubMed Abstract | 10.1186/s13550-019-0550-5 | Google Scholar 31440854 PMC6706501

[B109] Saam T. Ferguson M. S. Yarnykh V. L. Takaya N. Xu D. Polissar N. L. (2005). Quantitative evaluation of carotid plaque composition by *in vivo* MRI. Arterioscler. Thromb. Vasc. Biol. 25 (1), 234–239. 10.1161/01.ATV.0000149867.61851.31 PubMed Abstract | 10.1161/01.ATV.0000149867.61851.31 | Google Scholar 15528475

[B110] Saba L. Yuan C. Hatsukami T. S. Balu N. Qiao Y. DeMarco J. K. (2018). Carotid artery wall imaging: Perspective and guidelines from the ASNR vessel wall imaging study group and expert consensus recommendations of the American society of Neuroradiology. AJNR. Am. J. Neuroradiol. 39 (2), E9-E31. 10.3174/ajnr.A5488 PubMed Abstract | 10.3174/ajnr.A5488 | Google Scholar 29326139 PMC7410574

[B111] Sanz J. Fayad Z. A. (2008). Imaging of atherosclerotic cardiovascular disease. Nature 451 (7181), 953–957. 10.1038/nature06803 PubMed Abstract | 10.1038/nature06803 | Google Scholar 18288186

[B112] Segers F. Yu H. Molenaar T. Prince P. Tanaka T. Berkel T. V. (2012). Design and validation of a specific scavenger receptor class AI binding peptide for targeting the inflammatory atherosclerotic plaque. Arterioscler. Thromb. Vasc. Biol. 32 (4), 971–978. 10.1161/ATVBAHA.111.235358 PubMed Abstract | 10.1161/ATVBAHA.111.235358 | Google Scholar 22282357

[B113] Senders M. L. Hernot S. Carlucci G. Van D. Fay F. Calcagno C. (2018). Nanobody−facilitated multiparametric PET/MRI phenotyping of atherosclerosis. JACC. Cardiovasc. Imaging 12 (10), 2015–2026. 10.1016/j.jcmg.2018.07.027 PubMed Abstract | 10.1016/j.jcmg.2018.07.027 | Google Scholar 30343086 PMC6461528

[B114] Shah P. K. (2009). Inflammation and plaque vulnerability. Cardiovasc. Drugs Ther. 23 (1), 31–40. 10.1007/s10557-008-6147-2 PubMed Abstract | 10.1007/s10557-008-6147-2 | Google Scholar 18949542

[B115] Shan X. Zhang C. Mai C. Hu X. Xie Y. Chen W. (2021). The biogenesis, biological functions, and applications of Macrophage−Derived exosomes. Front. Mol. Biosci. 8, 715461. 10.3389/fmolb.2021.715461 PubMed Abstract | 10.3389/fmolb.2021.715461 | Google Scholar 34368234 PMC8333870

[B116] Shi C. Xie H. Ma Y. Yang Z. Zhang J. (2020). Nanoscale technologies in highly sensitive diagnosis of cardiovascular diseases. Front. Bioeng. Biotechnol. 8, 531. 10.3389/fbioe.2020.00531 PubMed Abstract | 10.3389/fbioe.2020.00531 | Google Scholar 32582663 PMC7289988

[B117] Shinohara M. Yamashita T. Tawa H. Takeda M. Sasaki N. Takaya T. (2008). Atherosclerotic plaque imaging using phase-contrast X−ray computed tomography. Am. J. Physiol. Heart Circ. Physiol. 294 (2), H1094–H1100. 10.1152/ajpheart.01149.2007 PubMed Abstract | 10.1152/ajpheart.01149.2007 | Google Scholar 18083896

[B118] Si−Mohamed S. A. Sigovan M. Hsu J. C. Tatard−Leitman V. Chalabreysse L. Naha P. C. (2021). *In vivo* molecular K−Edge imaging of atherosclerotic plaque using photon−counting ct. Radiology 300 (1), 98–107. 10.1148/radiol.2021203968 PubMed Abstract | 10.1148/radiol.2021203968 | Google Scholar 33944628 PMC8217298

[B119] Smith B. R. Heverhagen J. Knopp M. Schmalbrock P. Shapiro J. Shiomi M. (2007). Localization to atherosclerotic plaque and biodistribution of biochemically derivatized superparamagnetic iron oxide nanoparticles (SPIONs) contrast particles for magnetic resonance imaging (MRI). Biomed. Microdevices 9 (1), 719–727. 10.1007/s10544-007-9081-3 PubMed Abstract | 10.1007/s10544-007-9081-3 | Google Scholar 17562181

[B120] Soehnlein O. Libby P. (2021). Targeting inflammation in atherosclerosis — From experimental insights to the clinic. Nat. Rev. Drug Discov. 20 (8), 589–610. 10.1038/s41573-021-00198-1 PubMed Abstract | 10.1038/s41573-021-00198-1 | Google Scholar 33976384 PMC8112476

[B121] Song C. Labhasetwar V. Cui X. Underwood T. Levy R. J. (1998). Arterial uptake of biodegradable nanoparticles for intravascular local drug delivery: Results with an acute dog model. J. Control. Release 54 (2), 201–211. 10.1016/s0168-3659(98)00016-9 PubMed Abstract | 10.1016/s0168-3659(98)00016-9 | Google Scholar 9724907

[B122] Steinberg I. Huland D. M. Vermesh O. Frostig H. E. Tummers W. S. Gambhir S. S. (2019). Photoacoustic clinical imaging. Photoacoustics 14, 77–98. 10.1016/j.pacs.2019.05.001 PubMed Abstract | 10.1016/j.pacs.2019.05.001 | Google Scholar 31293884 PMC6595011

[B123] Stein−Merlob A. F. Hara T. Mccarthy J. R. Mauskapf A. Jaffer F. A. (2017). Atheroma susceptible to thrombosis exhibit impaired endothelial permeability *in vivo* as assessed by nanoparticle−based fluorescence molecular imaging clinical perspective. Circ. Cardiovasc Imag. 10 (5), e005813. 10.1161/CIRCIMAGING.116.005813 10.1161/CIRCIMAGING.116.005813 | Google Scholar PMC5509162 28487316

[B124] Stöger J. Gijbels M. J. J. Velden S. Manca M. Winther M. P. J. D. Biessen E. A. L. (2012). Distribution of macrophage polarization markers in human atherosclerosis. Atherosclerosis 225 (2), 461–468. 10.1016/j.atherosclerosis.2012.09.013 PubMed Abstract | 10.1016/j.atherosclerosis.2012.09.013 | Google Scholar 23078881

[B125] Ta H. T. Li Z. Hagemeyer C. E. Cowin G. Zhang S. Palasubramaniam J. (2017). Molecular imaging of activated platelets via antibody−targeted ultra−small iron oxide nanoparticles displaying unique dual MRI contrast. Biomaterials 134, 31–42. 10.1016/j.biomaterials.2017.04.037 PubMed Abstract | 10.1016/j.biomaterials.2017.04.037 | Google Scholar 28453956

[B126] Ta H. T. Nina A. Yuao W. Jean L. H. Shea L. Run Z. (2018). Activatable magnetic resonance nanosensor as a potential imaging agent for detecting and discriminating thrombosis. Nanoscale 10 (31), 15103–15115. 10.1039/c8nr05095c PubMed Abstract | 10.1039/c8nr05095c | Google Scholar 30059122

[B127] Tabas I. Williams K. J. Boren J. (2007). Subendothelial lipoprotein retention as the initiating process in atherosclerosis: Update and therapeutic implications. Circulation 116 (16), 1832–1844. 10.1161/CIRCULATIONAHA.106.676890 PubMed Abstract | 10.1161/CIRCULATIONAHA.106.676890 | Google Scholar 17938300

[B128] Tang J. Baxter S. Menon A. Alaarg A. Sanchezgaytan B. L. Fay F. (2016). Immune cell screening of a nanoparticle library improves atherosclerosis therapy. Proc. Natl. Acad. Sci. U. S. A. 113 (44), E6731-E6740. 10.1073/pnas.1609629113 PubMed Abstract | 10.1073/pnas.1609629113 | Google Scholar 27791119 PMC5098679

[B129] Tang T. Y. Howarth S. Miller S. R. Graves M. J. Patterson A. J. U−King−Im J. M. (2009). The ATHEROMA (Atorvastatin Therapy: Effects on Reduction of Macrophage Activity) Study. Evaluation using ultrasmall superparamagnetic iron oxide−enhanced magnetic resonance imaging in carotid disease. J. Am. Coll. Cardiol. 53 (22), 2039–2050. 10.1016/j.jacc.2009.03.018 PubMed Abstract | 10.1016/j.jacc.2009.03.018 | Google Scholar 19477353

[B130] Tang T. Y. Howarth S. Miller S. R. Graves M. J. U−King−Im J. M. Li Z. Y. (2008). Comparison of the inflammatory burden of truly asymptomatic carotid atheroma with atherosclerotic plaques in patients with asymptomatic carotid stenosis undergoing coronary artery bypass grafting: An ultrasmall superparamagnetic iron oxide enhanced magnetic resonance study. Eur. J. Vasc. Endovasc. Surg. 35 (4), 392–398. 10.1016/j.ejvs.2007.10.019 PubMed Abstract | 10.1016/j.ejvs.2007.10.019 | Google Scholar 18171628

[B131] Tarin C. Carril M. Martin−Ventura J. L. Markuerkiaga I. Padro D. Llamas−Granda P. (2015). Targeted gold−coated iron oxide nanoparticles for CD163 detection in atherosclerosis by MRI. Sci. Rep. 5, 17135. 10.1038/srep17135 PubMed Abstract | 10.1038/srep17135 | Google Scholar 26616677 PMC4663748

[B132] Tarkin J. M. Joshi F. R. Rudd J. (2014). PET imaging of inflammation in atherosclerosis. Nat. Rev. Cardiol. 11 (8), 443–457. 10.1038/nrcardio.2014.80 PubMed Abstract | 10.1038/nrcardio.2014.80 | Google Scholar 24913061

[B133] Tawakol A. Fayad Z. A. Mogg R. Alon A. Klimas M. T. Dansky H. (2013). Intensification of statin therapy results in a rapid reduction in atherosclerotic inflammation: Results of a multicenter fluorodeoxyglucose−positron emission tomography/computed tomography feasibility study. J. Am. Coll. Cardiol. 62 (10), 909–917. 10.1016/j.jacc.2013.04.066 PubMed Abstract | 10.1016/j.jacc.2013.04.066 | Google Scholar 23727083

[B134] Tawakol A. Ishai A. Takx R. A. Figueroa A. L. Ali A. Kaiser Y. (2017). Relation between resting amygdalar activity and cardiovascular events: A longitudinal and cohort study. Lancet 389, 834–845. 10.1016/S0140-6736(16)31714-7 PubMed Abstract | 10.1016/S0140-6736(16)31714-7 | Google Scholar 28088338 PMC7864285

[B135] Tawakol A. Migrino R. Q. Bashian G. G. Bedri S. Vermylen D. Cury R. C. (2006). *In vivo* ^18^F−fluorodeoxyglucose positron emission tomography imaging provides a noninvasive measure of carotid plaque inflammation in patients. J. Am. Coll. Cardiol. 48 (9), 1818–1824. 10.1016/j.jacc.2006.05.076 PubMed Abstract | 10.1016/j.jacc.2006.05.076 | Google Scholar 17084256

[B136] Terashima M. Uchida M. Kosuge H. Tsao P. S. Young M. J. Conolly S. M. (2011). Human ferritin cages for imaging vascular macrophages. Biomaterials 32 (5), 1430–1437. 10.1016/j.biomaterials.2010.09.029 PubMed Abstract | 10.1016/j.biomaterials.2010.09.029 | Google Scholar 21074263 PMC3012961

[B137] Thackeray J. T. Derlin T. Haghikia A. Napp L. C. Wang Y. Ross T. L. (2015). Molecular imaging of the chemokine receptor CXCR4 after acute myocardial infarction. JACC. Cardiovasc. Imaging 8 (12), 1417–1426. 10.1016/j.jcmg.2015.09.008 PubMed Abstract | 10.1016/j.jcmg.2015.09.008 | Google Scholar 26577262

[B138] Thapa N. Hong H. Y. Sangeetha P. Kim I. S. Yoo J. Rhee K. (2008). Identification of a peptide ligand recognizing dysfunctional endothelial cells for targeting atherosclerosis. J. Control. Release 131 (1), 27–33. 10.1016/j.jconrel.2008.07.013 PubMed Abstract | 10.1016/j.jconrel.2008.07.013 | Google Scholar 18680772

[B139] Thayse K. Kindt N. Laurent S. Carlier S. (2020). VCAM−1 target in non-invasive imaging for the detection of atherosclerotic plaques. Biol. (Basel) 9 (11), 368. 10.3390/biology9110368 10.3390/biology9110368 | Google Scholar PMC7692297 33138124

[B140] Tiwari A. Elgrably B. Saar G. Vandoorne K. (2021). Multi−Scale imaging of vascular pathologies in cardiovascular disease. Front. Med. 8, 754369. 10.3389/fmed.2021.754369 10.3389/fmed.2021.754369 | Google Scholar PMC8766766 35071257

[B141] van Tilborg G. A. Vucic E. Strijkers G. J. Cormode D. P. Mani V. Skajaa T. (2010). Annexin A5−functionalized bimodal nanoparticles for MRI and fluorescence imaging of atherosclerotic plaques. Bioconjug. Chem. 21 (10), 1794–1803. 10.1021/bc100091q PubMed Abstract | 10.1021/bc100091q | Google Scholar 20804153 PMC3190195

[B142] Virmani R. Burke A. P. Farb A. Kolodgie F. D. (2006). Pathology of the vulnerable plaque. J. Am. Coll. Cardiol. 47, C13–C18. 10.1016/j.jacc.2005.10.065 PubMed Abstract | 10.1016/j.jacc.2005.10.065 | Google Scholar 16631505

[B143] Wang J. Liu J. Liu Y. Wang L. Cao M. Ji Y. (2016). Gd−Hybridized plasmonic Au−Nanocomposites enhanced Tumor−Interior drug permeability in multimodal Imaging−Guided therapy. Adv. Mat. 28 (40), 8950–8958. 10.1002/adma.201603114 PubMed Abstract | 10.1002/adma.201603114 | Google Scholar 27562240

[B144] Wang J. Wu M. Chang J. Li L. Guo Q. Hao J. (2019). Scavenger receptor−AI−targeted ultrasmall gold nanoclusters facilitate *in vivo* MR and *ex vivo* fluorescence dual−modality visualization of vulnerable atherosclerotic plaques. Nanomedicine 19, 81–94. 10.1016/j.nano.2019.04.003 PubMed Abstract | 10.1016/j.nano.2019.04.003 | Google Scholar 31028886

[B145] Wang X. Searle A. K. Hohmann J. D. Liu A. L. Abraham M. K. Palasubramaniam J. (2018). Dual−targeted theranostic delivery of miRs arrests abdominal aortic aneurysm development. Mol. Ther. 26 (4), 1056–1065. 10.1016/j.ymthe.2018.02.010 PubMed Abstract | 10.1016/j.ymthe.2018.02.010 | Google Scholar 29525742 PMC6080135

[B146] Wang Y. Zhang K. Qin X. Li T. Qiu J. Yin T. (2019). Biomimetic nanotherapies: Red blood cell based core–shell structured nanocomplexes for atherosclerosis management. Adv. Sci. 6 (12), 1900172. 10.1002/advs.201900172 10.1002/advs.201900172 | Google Scholar PMC6662054 31380165

[B147] Wang Y. Zhang Y. Wang Z. Zhang J. Qiao R. R. Xu M. (2019). Optical/MRI dual−modality imaging of M1 macrophage polarization in atherosclerotic plaque with MARCO−targeted upconversion luminescence probe. Biomaterials 219, 119378. 10.1016/j.biomaterials.2019.119378 PubMed Abstract | 10.1016/j.biomaterials.2019.119378 | Google Scholar 31382209

[B148] Wang Z. Wang X. Wan J. B. Xu F. Zhao N. Chen M. (2021). Optical imaging in the second near infrared window for vascular bioimaging. Small 17 (43), e2103780. 10.1002/smll.202103780 PubMed Abstract | 10.1002/smll.202103780 | Google Scholar 34643028

[B149] Wehrse E. Klein L. Rotkopf L. T. Wagner W. L. Uhrig M. Heußel C. P. (2021). Photon−counting detectors in computed tomography: From quantum physics to clinical practice. Radiologe 61 (1), 1–10. 10.1007/s00117-021-00812-8 10.1007/s00117-021-00812-8 | Google Scholar 33598788

[B150] Wei C. Vucic E. Leupold E. Mulder W. Cormode D. P. Briley−Saebo K. C. (2010). Incorporation of an apoE−derived lipopeptide in high−density lipoprotein MRI contrast agents for enhanced imaging of macrophages in atherosclerosis. Contrast Media Mol. Imaging 3 (6), 233–242. 10.1002/cmmi.257 10.1002/cmmi.257 | Google Scholar 19072768

[B151] Wei X. Ying M. Dehaini D. Su Y. Kroll A. V. Zhou J. (2018). Nanoparticle functionalization with platelet membrane enables multifactored biological targeting and detection of atherosclerosis. ACS Nano 12 (1), 109–116. 10.1021/acsnano.7b07720 PubMed Abstract | 10.1021/acsnano.7b07720 | Google Scholar 29216423 PMC5859122

[B152] Weller G. E. R. Villanueva F. S. Tom E. M. Wagner W. R. (2005). Targeted ultrasound contrast agents: *In vitro* assessment of endothelial dysfunction and multi−targeting to ICAM−1 and sialyl lewisx. Biotechnol. Bioeng. 92 (6), 780–788. 10.1002/bit.20625 PubMed Abstract | 10.1002/bit.20625 | Google Scholar 16121392

[B153] Wen S. Liu D. F. Cui Y. Harris S. S. Chen Y. C. Li K. C. (2014). *In vivo* MRI detection of carotid atherosclerotic lesions and kidney inflammation in ApoE−deficient mice by using LOX−1 targeted iron nanoparticles. Nanomedicine 10 (3), 639–649. 10.1016/j.nano.2013.09.009 PubMed Abstract | 10.1016/j.nano.2013.09.009 | Google Scholar 24103305

[B154] Wu J. Leong−Poi H. Bin J. Yang L. Liao Y. Liu Y. (2011). Efficacy of contrast−enhanced US and magnetic microbubbles targeted to vascular cell adhesion molecule−1 for molecular imaging of atherosclerosis. Radiology 260 (2), 463–471. 10.1148/radiol.11102251 PubMed Abstract | 10.1148/radiol.11102251 | Google Scholar 21555346

[B155] Wu M. Li X. Guo Q. Li J. Xu G. Li G. (2021). Magnetic mesoporous silica nanoparticles−aided dual MR/NIRF imaging to identify macrophage enrichment in atherosclerotic plaques. Nanomedicine 32, 102330. 10.1016/j.nano.2020.102330 PubMed Abstract | 10.1016/j.nano.2020.102330 | Google Scholar 33171287

[B156] Xing H. Zhang S. Bu W. Zheng X. Wang L. Xiao Q. (2014). Ultrasmall NaGdF_4_ nanodots for efficient MR angiography and atherosclerotic plaque imaging. Adv. Mat. 26 (23), 3867–3872. 10.1002/adma.201305222 PubMed Abstract | 10.1002/adma.201305222 | Google Scholar 24677351

[B157] Yang X. Li J. Hu D. Chen J. Li Y. Huang J. (2016). Predicting the 10−Year risks of atherosclerotic cardiovascular disease in Chinese population: The China−PAR project (prediction for ASCVD risk in China). Circulation 134 (19), 1430–1440. 10.1161/CIRCULATIONAHA.116.022367 PubMed Abstract | 10.1161/CIRCULATIONAHA.116.022367 | Google Scholar 27682885

[B158] Yao J. Yang Z. Huang L. Yang C. Wang J. Cao Y. (2021). Low−intensity focused ultrasound−responsive ferrite−encapsulated nanoparticles for atherosclerotic plaque neovascularization theranostics. Adv. Sci. 8 (19), e2100850. 10.1002/advs.202100850 10.1002/advs.202100850 | Google Scholar PMC8498883 34382370

[B159] Yurdagul A. Jr. Subramanian M. Wang X. Crown S. B. Ilkayeva O. R. Darville L. (2020). Macrophage metabolism of apoptotic Cell−Derived arginine promotes continual efferocytosis and resolution of injury. Cell. Metab. 31 (3), 518518–518533. 10.1016/j.cmet.2020.01.001 10.1016/j.cmet.2020.01.001 | Google Scholar PMC7173557 32004476

[B160] Zhang L. Xue S. Ren F. Huang S. Zhou R. Wang Y. (2021). An atherosclerotic plaque−targeted single−chain antibody for MR/NIR−II imaging of atherosclerosis and anti−atherosclerosis therapy. J. Nanobiotechnology 19 (1), 296. 10.1186/s12951-021-01047-4 PubMed Abstract | 10.1186/s12951-021-01047-4 | Google Scholar 34583680 PMC8479957

[B161] Zhao D. Liu J. Wang M. Zhang X. Zhou M. (2019). Epidemiology of cardiovascular disease in China: Current features and implications. Nat. Rev. Cardiol. 16 (4), 203–212. 10.1038/s41569-018-0119-4 PubMed Abstract | 10.1038/s41569-018-0119-4 | Google Scholar 30467329

[B162] Zheng K. H. Schoormans J. Stiekema L. C. A. Calcagno C. Cicha I. Alexiou C. (2019). Plaque permeability assessed with DCE−MRI associates with uspio uptake inpatients with peripheral artery disease. JACC. Cardiovasc. Imaging 12 (10), 2081–2083. 10.1016/j.jcmg.2019.04.014 PubMed Abstract | 10.1016/j.jcmg.2019.04.014 | Google Scholar 31202746

